# Identification of a Novel Polyamine Scaffold With Potent Efflux Pump Inhibition Activity Toward Multi-Drug Resistant Bacterial Pathogens

**DOI:** 10.3389/fmicb.2018.01301

**Published:** 2018-06-14

**Authors:** Renee M. Fleeman, Ginamarie Debevec, Kirsten Antonen, Jessie L. Adams, Radleigh G. Santos, Gregory S. Welmaker, Richard A. Houghten, Marc A. Giulianotti, Lindsey N. Shaw

**Affiliations:** ^1^Department of Cell Biology, Microbiology and Molecular Biology, University of South Florida, Tampa, FL, United States; ^2^Torrey Pines Institute for Molecular Studies, Port St. Lucie, FL, United States

**Keywords:** efflux pumps, efflux pump inhibitors, polyamines, bacterial resistance, potentiation

## Abstract

We have previously reported the use of combinatorial chemistry to identify broad-spectrum antibacterial agents. Herein, we extend our analysis of this technology toward the discovery of anti-resistance molecules, focusing on efflux pump inhibitors. Using high-throughput screening against multi-drug resistant *Pseudomonas aeruginosa*, we identified a polyamine scaffold that demonstrated strong efflux pump inhibition without possessing antibacterial effects. We determined that these molecules were most effective with an amine functionality at R1 and benzene functionalities at R2 and R3. From a library of 188 compounds, we studied the properties of 5 lead agents in detail, observing a fivefold to eightfold decrease in the 90% effective concentration of tetracycline, chloramphenicol, and aztreonam toward *P. aeruginosa* isolates. Additionally, we determined that our molecules were not only active toward *P. aeruginosa*, but toward *Acinetobacter baumannii* and *Staphylococcus aureus* as well. The specificity of our molecules to efflux pump inhibition was confirmed using ethidium bromide accumulation assays, and in studies with strains that displayed varying abilities in their efflux potential. When assessing off target effects we observed no disruption of bacterial membrane polarity, no general toxicity toward mammalian cells, and no inhibition of calcium channel activity in human kidney cells. Finally, combination treatment with our lead agents engendered a marked increase in the bactericidal capacity of tetracycline, and significantly decreased viability within *P. aeruginosa* biofilms. As such, we report a unique polyamine scaffold that has strong potential for the future development of novel and broadly active efflux pump inhibitors targeting multi-drug resistant bacterial infections.

## Introduction

The continued increase of antimicrobial resistant bacterial infections is an ongoing public health crisis in the United States, with mortality rates increasing yearly (Boucher et al., 2009). This problem can be directly linked to ever growing demands for antibiotics, coupled with a diminishing therapeutic arsenal that has been exacerbated by a continual decline in antibiotic discovery over the past 30 years (Spellberg et al., 2008). This presents the scenario of a much talked about post-antibiotic era, where conventional antibiotics will no longer be effective and common infections may once again become fatal (Reardon, 2014). A primary issue is that typical drug discovery efforts often result in the development of therapeutics with known mechanisms of action, thus allowing bacteria to rapidly evolve resistance to these new agents (Falagas and Bliziotis, 2007). Consequently, new strategies are urgently needed for the discovery of novel therapeutics targeting multi-drug resistant organisms (Spellberg et al., 2008; Reardon, 2014).

The selective pressure antibiotics place on heterogeneous bacterial communities often directly leads to resistant clones becoming dominant within infectious populations (Spellberg et al., 2008). Novel therapeutics targeting resistant bacterial strains would be therapeutically advantageous, specifically focusing on those isolates that prove the most difficult to eradicate. A unique way to do this is to interfere with bacterial resistance mechanisms, rather than focusing on bacterial viability. Such treatment options could restore the effectiveness of numerous obsolete clinical antibiotics, reclaiming many important therapeutics. Co-administration of such anti-resistance agents alongside existing antibiotics may also lead to decreased resistance, as multiple targets within the cell are impacted simultaneously (Yoneyama and Katsumata, 2014). Hence, anti-resistance approaches may exponentially increase the number of available therapeutic options, whereas conventional antibiotic development commonly yield only a single new drug (Renau et al., 1999; Lomovskaya et al., 2001).

A primary method by which bacteria resist the action of antibacterial agents is via efflux pump extrusion. Efflux pumps are complexes within the bacterial cell envelope used to export toxic substances such as antibiotics from the intracellular environment before damage to the cell occurs (Sun et al., 2014). Efflux pumps are found in most multi-drug resistant nosocomial pathogens, with many having similar and overlapping substrate specificities. As such, targeting bacterial efflux pumps via therapeutic intervention could effectively re-sensitize cells to a broad spectrum of antibacterial agents (Renau et al., 1999; Lomovskaya et al., 2001; Weinstein and Hooper, 2005). Indeed, recent studies have shown that strains overexpressing efflux pumps commonly display an average >twofold increased minimal inhibitory concentrations (MIC) toward multiple antibiotics (Weinstein and Hooper, 2005; Adabi et al., 2015).

Efflux mediated resistance was first described almost 40 years ago in a study demonstrating that tetracycline insensitivity could result from plasmid-encoded transport systems (McMurry et al., 1980). Following this, Nelson et al. (1993, 1994) and Nelson and Levy (1999) observed that polyamine tetracycline derivatives could increase the effectiveness of tetracycline when administered concomitantly. Early inhibitors targeting efflux pumps, such as reserpine, were discovered from existing drugs, however their use was limited by the need for administration at very high doses in order to be effective (Van Bambeke et al., 2006). They also suffered from off-target effects, with compounds such as verapamil, reserpine, and thioridazine not only inhibiting bacterial efflux pumps, but eukaryotic transporters as well (Cornwell et al., 1987; Neyrolles et al., 2016). Specifically, molecules such as verapamil when administered at higher doses have been shown to cause cardiac arrest due to calcium channel inhibition (Chevalier et al., 1999). More recent agents, such as MC-207,110 (phenylalanine arginine beta naphthalimide, or PaβN), have been shown to have increased specificity toward bacterial efflux systems, (Renau et al., 1999; Lomovskaya et al., 2001; Misra and Bavro, 2009; Opperman and Nguyen, 2015); however, the advancement of this scaffold has been abandoned as it has been shown to non-specifically depolarize prokaryotic membranes and cause significant nephrotoxicity (Watkins et al., 2003; Webber et al., 2013; Opperman and Nguyen, 2015). Although a number of efflux pump inhibitors with improved activity have been identified in recent years (Chamberland et al., 1996, 1999, 2000, 2001; Zelle and Harding, 1996; Levy, 1998; Zelle, 1998; Markham et al., 2000; De Souza et al., 2002; Nakayama et al., 2002; Pages et al., 2003; Nelson and Alekhsun, 2004; Lemaire et al., 2006), the only advancement into clinical trials to date has been for the proton pump inhibitor omeprazole, used in combination with amoxicillin and clarithromycin to treat *Helicobacter pylori* infections (Handzlik et al., 2013; ClinicalTrials.gov, 2014). Therefore, there is a clear need to identify new efflux pump inhibitors with enhanced properties, and limited toxicity. This is particularly true for Gram negative species, such as *Pseudomonas aeruginosa*, which have impermeable outer membranes and commonly overexpress efflux systems (Palmer and Whiteley, 2015; Tommasi et al., 2015). Indeed, this organism has 10 resistance nodulation division (RND) efflux pumps that collectively extrude β-lactams, fluoroquinolones, sodium dodecyl sulfate, tetracycline, erythromycin, ethidium bromide, crystal violet, and homoserine lactones (Askoura et al., 2011). Moreover, given the broad substrate range of *P. aeruginosa* efflux pumps, the inhibition of one pump can be alleviated by the upregulation of additional efflux pumps with parallel targets (Lister et al., 2009).

In previous work by our group we used combinatorial chemistry to identify broad spectrum antibacterial agents (Fleeman et al., 2015; Sandhaus et al., 2016). In the present study, we extend our analysis of this technology toward the discovery of anti-resistance agents, specifically focusing on efflux pump inhibitors. Using high throughput combinatorial scaffold library screening against multi-drug resistant *P. aeruginosa* isolates we identified a polyamine scaffold derived from a reduced acyl peptide that demonstrated strong efflux pump inhibition and limited cytotoxicity toward eukaryotic cells. We suggest that these molecules possess excellent potential for future development as anti-resistance agents targeting bacterial efflux pumps.

## Materials and Methods

### Design and Synthesis of the Combinatorial Libraries

The design and synthesis of the Torrey Pines scaffold ranking library has previously been described (Houghten et al., 1999; Reilley et al., 2010; Santos et al., 2013; Wu et al., 2013). The library is comprised of 84 different scaffolds, each with 10,000–750,000 compounds, in approximately equal molar amounts. The polyamine library chosen for analysis contains 399,766 analogs; from this, 188 individual compounds were chosen for analysis. Detailed chemical characterization for scaffold libraries and individual compounds can be found in the general chemistry method section in Supplementary Figure S1. Individual compounds were synthesized as described in Scheme a in Supplementary Figure S1.

### Bacterial Strains and Growth Conditions

The bacterial strains used in this study are multi-drug resistant clinical isolates that have previously been described (Supplementary Table S1; Fleeman et al., 2015). Organisms were grown in tryptic soy broth (TSB) for overnight cultures and cation adjusted Mueller Hinton broth (CA-MH II) was used for experimental procedures. All incubations were performed at 37°C. The minimum inhibitory concentrations (MICS) for all antibiotics tested against these organisms are shown in Supplementary Table S1.

### Checkerboard Potentiation Assays

Scaffold ranking library samples and individual polyamines were screened using checkerboard inhibitory assays to assess the potentiation of tetracycline and chloramphenicol. The test utilized a 96-well plate microtiter assay where the concentration of the scaffold or individual polyamine was decreased from 25 to 0.8 μg mL^-1^ (average molarity of mixture samples 65 to 4 μM) along the rows, and the concentration of tetracycline or chloramphenicol was increased from 0.4 to 100 μM across the columns. The EC_90_ values for all polyamines are reported in μg mL^-1^ which more accurately displays the effective concentrations for mixture samples. However, with the known antibiotics, we report EC_90_ values in μM, which more accurately describes purified compounds. Plates were incubated statically at 37°C for 20 h, and the optical density (OD_600_) was determined using a Synergy 2 plate reader (BioTek). Potentiation modeling (detailed below) was performed to determine fold change in the 50 and 90% effective concentration of tetracycline or chloramphenicol.

### Statistical Analysis of Checkerboard Assays

Potentiation was quantified using a mathematical model developed by our group to assess the ability of library samples and individual compounds to effectively enhance the activity of tetracycline or chloramphenicol (Hoel, 1987). This was used to differentiate libraries or compounds that possessed only antibacterial activity from those that had synergistic activity with tetracycline or chloramphenicol. In this way, only libraries and compounds that potentiated tetracycline or chloramphenicol activity were pursued. The model is based on the following equation for modeling the percentage activity of a mixture of two agents with independent action (Hoel, 1987):

$${\%}_{\text{Antibiotic }\&\text{ Comp}}\left(X_{1},X_{2}\right)={\%}_{\text{Antibiotic}}\left(X_{1}\right)+{\%}_{\text{Comp}}(X_{2})-{\%}_{\text{Antibiotic}}\left(X_{1}\right)\cdot {\%}_{\text{Comp}}(X_{2})$$

Here, x_1_ and x_2_ are the concentrations of antibiotic (tetracycline or chloramphenicol) and library/compound (Comp) tested, respectively. This equation can be rearranged to model the effective percent activity (EC) of the antibiotic alone, after accounting for compound activity:

$${Eff\%}_{Antibiotic}(X_{1})=\frac{{\%}_{\text{Antibiotic }\&\text{ Compound}}(X_{1},X_{2})-{\%}_{\text{Compound}}\left(X_{2}\right)}{1-{\%}_{Compound}(X_{2})}$$

Thus, the model-adjusted checkerboards show the antibiotic activity post-potentiation, and from that one can determine the true change in Mic.

### Ethidium Bromide Efflux Inhibition Assay

Ethidium bromide efflux assessment was performed by following the fluorescence of ethidium bromide in a 96-well plate assay, as described previously (Renau et al., 1999; Lomovskaya et al., 2001; Webber et al., 2013; Blanchard et al., 2014; Vasudevan et al., 2014). This varies from ethidium bromide accumulation assays as it includes a pre-incubation step to allow accumulation before assessment of efflux (Raherison et al., 2002; Paixao et al., 2009). Bacterial cells were grown overnight at 37°C in TSB, before being synchronized for 3 h in fresh media to exponential phase. Cultures were pelleted at 900 × *g* for 20 min and the supernatant removed. The resulting pellet was thrice washed and resuspended in 20 mM sodium phosphate buffer to an OD_600_ of 0.2. Ethidium bromide was next added at a sub inhibitory concentration of 25 μM and incubated at room temperature for 15 min to equilibrate. After equilibration, cells were inoculated into a black walled 96-well plate to a density of 1 × 10^6^ CFU mL^-1^. Using a Biotek plate reader, the fluorescence of cells was monitored for 2 min with 530 nm excitation and 600 nm emission. When baseline readings were complete, polyamines 247, 250, 266, 271, and 314 were added at 25 μg mL^-1^ alongside the positive control PaβN at the same concentration [all efflux pumps inhibitor concentrations were maximum potentiating concentrations (MPC)]. The solvent dimethylformamide (DMF) was used as a no treatment control alongside tetracycline alone. After addition of all compounds, fluorescence was monitored every 5 min for a total of 90 min. After this time, cells were serially diluted and plated to ensure that treatment with ethidium bromide did not affect viability. There was an initial spike in fluorescence upon addition of the compound, with minimal changes observed thereafter, therefore for graphical representation, the final maximum relative fluorescence at 90 min was used for comparison of lead agents to controls.

### Bacterial Membrane Depolarization

To determine the level of membrane depolarization by polyamine compounds a 3,3′-dipropylthiadicarbocyanine Iodide (DiSC_3_) fluorescence dye was used. Exponentially growing cultures were prepared as described above, before being harvested by centrifugation. Cells were next washed in buffer a [5 mM Hepes (pH 7.2), 5 mM glucose] and resuspended to an OD_600_ of 0.2 in the same buffer containing 100 mM KCL and 2 μM DiSC_3_. Samples were allowed to equilibrate for 15 min at room temperature to ensure uptake and quenching of the dye in bacterial membranes. Cells were aliquoted into 96-well plates and polyamines were added alongside the known efflux inhibitor PAβN (all at 25 μg mL^-1^). Nisin (25 μg mL^-1^) was used as a positive control to display depolarization effects. A Biotek plate reader was used to monitor fluorescence of wells, with a 622 nm excitation and 670 nm emission. For graphical representation, the maximum relative fluorescence at 2 min was used for comparison of lead agents to controls.

### Eukaryotic Cell Cytotoxicity

To assess toxicity of polyamine compounds we used HepG2 human liver carcinoma cells and Hek293T human embryonic kidney epithelial cells. The viability of cell lines was determined using a 3-(4,5-dimethylthiazol-2-yl)-2,5-diphenyltetrazolium bromide (MTT) molecular probe as previously described by our group (Fleeman et al., 2015). Briefly, 247, 266, 271 (Hek293T), or 250, 271, 314 (HepG2), alongside control efflux pump inhibitor PaβN, were diluted in 10% DMF from 25 μg mL^-1^ to 3 μg mL^-1^ using twofold dilutions, before being added to cells in DMEM with 10% FBS and 1% penicillin/streptomycin. Cells were then incubated for 48 h at 37°C with 5% CO_2._ Following this, viability was assessed by the addition of MTT and measuring the OD_570_ in a Biotek plate reader. Percent recovery was determined compared to no drug controls.

### Eukaryotic Calcium Channel Activity Assay

The effects of polyamine efflux inhibitors on eukaryotic ion channels was performed using a calcium channel assay kit (Life Technologies) and the Hek293T kidney cell line. Cells (5 × 10^4^) were inoculated into a black walled 96-well plate and allowed to attach overnight at 37°C with 5% CO_2_. After this, the Fluo-4 dye supplemented with Probenecid (5 mM) was added and allowed to equilibrate for 1 h at 37°C with 5% CO_2_. After this time, fluorescence was measured for 120 s using a Biotek plate reader with a 495 nm excitation and 516 nm emission. Cells were then treated with solvent only controls (10% DMF), as well as polyamine compounds 250, 266, 271, and the known calcium channel inhibitor verapamil (all at 25 μg mL^-1^). Fluorescence was monitored for 120 s, before calcium channels were stimulated with carbamylcholine chloride (137 μM). Readings were then taken at 6 s intervals, with peak relative fluorescence at 18 s used graphically for comparison of lead agents to controls.

### Bactericidal and Biofilm Assessment

Lead agents were screened for bactericidal activity as described by us previously (Van Horn et al., 2014; Fleeman et al., 2015), with the following modifications. Varying concentrations of tetracycline (0, 12, 25, and 50 μM) were used in combination with the MPC (25 μM) of lead agents 247, 250, 266, 271, and 314 against *P. aeruginosa*. Data is shown as percent recovery by dividing the CFU mL^-1^ of treatment groups by the CFU mL^-1^ recovered from a no treatment control that did not have tetracycline or efflux inhibitors. Biofilm experiments were performed similar to those described by us previously (Fleeman et al., 2015; von Salm et al., 2016), with the following modifications. Polyamine agents 247, 250, 266, 271, and 314 were added at MPC into biofilm containing wells alongside varying concentrations of tetracycline (0, 12, 25, and 50 μM). Cellular viability was determined by serial dilution after a 20-h incubation at 37°C. Values were converted to percent recovery using no treatment controls. All data was generated from three biological replicates and two technical replicates.

## Results

### The Use of a Scaffold Ranking Library to Identify Potential Efflux Pump Inhibitors

**FIGURE 1:**
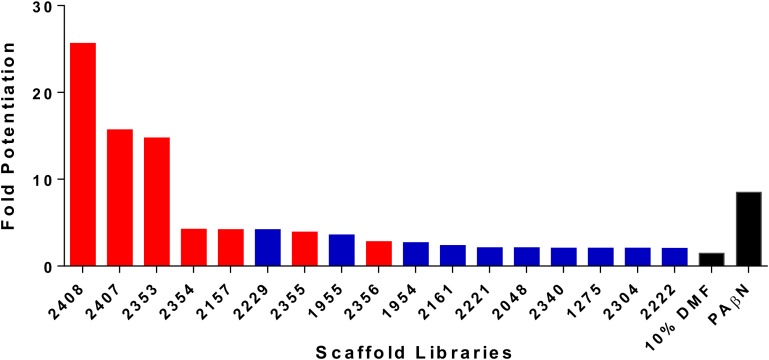
Screening of combinatorial libraries to identify scaffolds that inhibit bacterial efflux pumps. The Torrey Pines scaffold libraries (TPL) were screened for potentiation of tetracycline activity against a clinical tetracycline-resistant *P. aeruginosa* isolate 1419. Data is represented as fold potentiation, which is defined as the EC_90_ tetracycline concentration (no TPL)/EC_90_ tetracycline concentration with TPL. In each assay, the TPL concentration used was 25 μg mL^-1^. The libraries represented by a red bar displayed inhibition of bacterial growth themselves, in the absence of tetracycline, whilst blue bars represent libraries that display no inhibition of bacterial growth. Positive (PAβN) and negative (10% DMF) control compounds were used, and are denoted by black bars. Note that only libraries displaying twofold or greater potentiation are shown.

We have previously described the synthesis and antibacterial activity of the Torrey Pines scaffold ranking library toward the ESKAPE pathogens. With the success of this screening, we decided to expand our approaches toward the development of anti-resistance agents, specifically targeting efflux mechanisms (Fleeman et al., 2015). As such, the 81 Torrey Pines scaffold samples were screened for their ability to decrease the 90% effective concentration (EC_90_) of the known efflux pump substrate, tetracycline, toward a tetracycline resistant strain of *P. aeruginosa* (tetracycline alone EC_90_ = 82.5 μM). EC_90_s were defined as the concentration required to produce 90% inhibition of the test organism. The capacity for potentiation of all scaffolds was determined by mathematical modeling to identify (and thus exclude) those molecules that also possessed antibacterial activity themselves (see Materials and Methods section “Statistical Analysis of Checkerboard Assays” for details). Upon analysis, 17 libraries resulted in a potentiated tetracycline EC_90_ of ≥twofold, whilst 6 resulted in a fold potentiation of ≥4 (**Figure 1** and Supplementary Table S2). A consideration with these studies is that we wish to identify efflux pump inhibitors, rather than compounds that have bacterial killing activity themselves. Upon testing the top 17 libraries we determined that 7 of them, including the 5 most active scaffolds, had individual antibacterial activity. Of the remaining 10 libraries, 2229 (polyamines derived from reduced acyl peptides) had the best potentiating effects (>fourfold, tetracycline EC_90_ lowered to 21.03 μM), without itself having antibacterial activity. For this reason, we prioritized the 2229 polyamine scaffold for further assessment.

### Lead Polyamine Efflux Inhibitor Screening

To explore suitability of polyamine derivatives as efflux pump inhibitors, a library of 188 individual compounds contained within the Torrey Pines chemical collection were screened for their ability to decrease the 50 and 90% effective concentration of tetracycline toward *P. aeruginosa* (Supplementary Table S3). These studies were expanded to include EC_50_ determinations as well as EC_90_ to provide depth to our structure activity relationship analysis.

**FIGURE 2:**
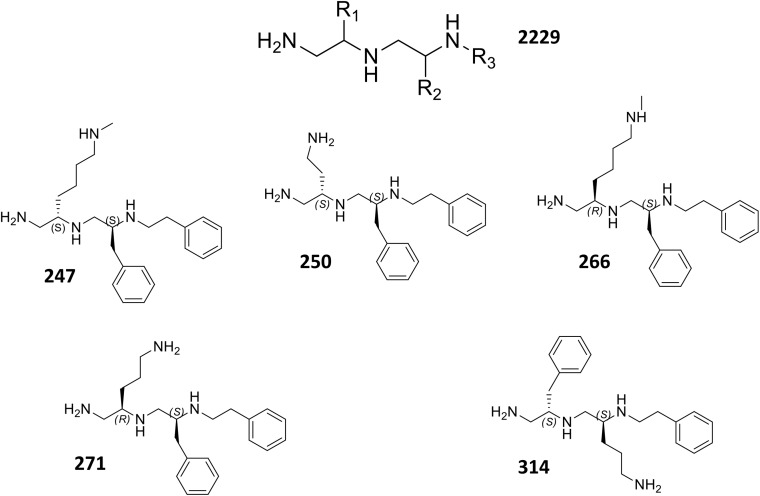
Structure of core polyamine scaffold (2229) and individual polyamine lead molecules.

Upon analysis, 37 individual polyamines were found to inhibit bacterial growth alone at or below the maximum concentration tested 25 μg mL^-1^. Of the 151 remaining polyamines, 72 reduced the tetracycline EC_50_ by <twofold, 30 decreased the tetracycline EC_50_ between twofold and fivefold, and 49 decreased the tetracycline EC_50_ by ≥fivefold. From this latter group, 10 were also successful at decreasing the 90% effective concentration by ≥fivefold. Four of the 10 most effective polyamines (**247**, **250**, **266**, **271**) had an amine functionality at the R1 position, *S*-methylbenzene at the R2 position, and ethylbenzene at the R3 position. Interestingly, both stereoisomers of methylbutylamine (**247** = *S-N*-methylbutylamine: **266** = *R-N*-methylbutylamine) were found to create strong potentiation at the R1 position. From the remaining six polyamines, three (**314**, **338**, and **348**) had *S*-methylbenzene at the R1 position, an amine functionality at the R2 position, and ethylbenzene at the R3 position; while three (**393**, **414**, and **453**) had *S*-methylbenzene at the R1 and R2, and varied aromatic groups at the R3 position; thus, lacking an amine functionality at the R1 or R2 position. Although polyamines **393**, **414**, and **453** displayed promising fold-potentiation values, these agents were not selected as lead agents when considering that a large portion (24%) of polyamines with the R1 and R2 functionality defined by *S*-methylbenzene displayed antibacterial activity themselves. In contrast, however, the majority (52%) of polyamines with *S*-methylbenzene at the R2 and R3 positions displayed ≥twofold potentiation of tetracycline activity without displaying inhibition alone. Therefore, we prioritized polyamines with amine functionalities at the R1 position (**247**, **250**, **266**, **271**), as this was the most promising orientation for the positive charge. In addition, while the 10 most potentiating polyamines were shown to decrease the EC_50_ of tetracycline from 47.8 μM to ≤9 μM, there were a subset of four agents (**247**, **250**, **271, 314**) that were more effective at decreasing the EC_90_ than the EC_50_ revealing their activity does not plateau before 90% bacterial inhibition is achieved. Therefore, we chose polyamines **247**, **250**, **266**, **271**, **314** from the 10 most potentiating polyamines to undergo secondary validation assessment to explore their efflux pump inhibitor-like properties (**Figure 2**).

**FIGURE 3:**
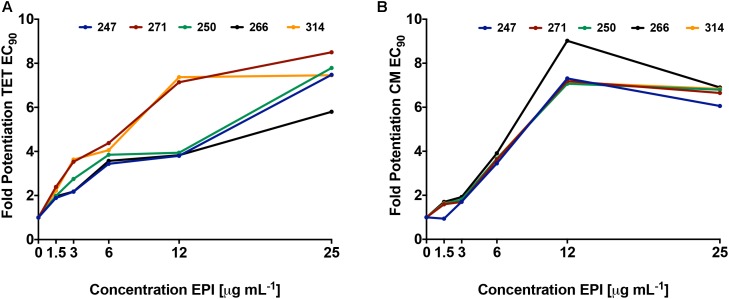
Polyamine lead agents potentiate the activity of unrelated antibiotic efflux substrates. *P. aeruginosa* (1419) cells were treated with polyamine molecules at increasing concentrations, alongside tetracycline **(A)** or chloramphenicol **(B)**. Shown is the fold potentiation of each antibiotic (EC_90_ values) as the efflux pump inhibitor concentration was increased.

Using dose response studies (**Figure 3A** and Supplementary Figure S2A), we determined that the most effective lead was compound **271**, potentiating the tetracycline EC_90_ by 8.5-fold and its EC_50_ by 8.2-fold (**Table 1**). With regards to the remaining four compounds, we determined that **247** resulted in an 8.3-fold decrease of the EC_90_ and a fivefold decrease of the EC_50_. Of note, these two compounds are similar with *S*-methylbenzene at the R2 position, and ethylbenzene at the R3 position, however, they differ slightly at the R1 position (**247** = *S-N*-methylbutylamine; **271** = *R-N*-propylamine). Additionally, compounds **250** and **266** both display a 7.8- and 5.8-fold potentiation of the tetracycline EC_90_, respectively, with strong EC_50_ values of 7.0- and 6.8-fold potentiation. Interestingly, **266** displayed more promising EC_50_ fold-potentiation than EC_90_, however this is a common feature of competitive efflux pump inhibitor (Askoura et al., 2011); indeed, our studies reveal a similar effect for the well described molecules reserpine and PAβN (**Table 1**). Both **250** and **266** also have *S*-methylbenzene at the R2 position and ethylbenzene at the R3 position similar to compounds **247** and **271**, but again vary at the R1 position (**250** = *S*-ethylamine; **266** = *R-N*-methylbutylamine). Compound **314** was found to have EC_50_ and EC_90,_ fold potentiation values of 5- and 7.5-fold, respectively. Of note, **314** has an *S*-methylbenzene at the R1 position and an amine functionality (this time propylamine) at the R2.

**Table 1:** Potentiation assessment of lead polyamine compounds.

	MW	EPI^∗a^	TET+EPI^∗b^	FP^∗c^	TET+EPI^#b^	FP^#c^
**247**	**368.56**	>25	9	5.0	12	8.3
**250**	**340.51**	>25	9	7.0	12	7.8
**266**	**368.56**	>25	9	6.8	16	5.8
**271**	**354.53**	>25	5	8.2	10	8.5
**314**	**354.53**	>25	9	5.0	12	7.5
**Reserpine**	**608.68**	>25	4	11.2	>50	1
**PAβN**	**519.47**	>25	0.7	65.5	11.63	8


**All assays were performed in triplicate alongside no drug controls, known efflux pump inhibitors (EPIs) reserpine and PAβN, and tetracycline alone controls (data not shown due to repetitive values, average concentration for EC_*50*_ across all replicates = 47.8 μM; EC_*90*_ = 82.5 μM). Molecular weight (MW) of all compounds is displayed as grams mole^-*1*^. ^∗^50% effective concentrations (EC_*50*_). ^#^90% effective concentrations (EC_*90*_). **^*a*^EPI** is the inhibitory concentration of the individual Efflux Pump Inhibitor (EPI) alone (μg mL^-*1*^).**^*b*^TET+EPI** is the respective EC tetracycline (μM) in the presence of each compound. ^*c*^**FP** is Fold Potentiation = EC tetracycline + EPI/EC tetracycline (no EPI).**

### Polyamines Inhibit Efflux Pump Activity for a Wide Range of Organisms and Antibiotic Substrates

**FIGURE 4:**
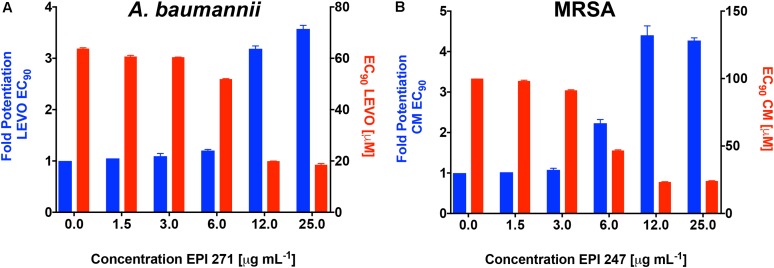
Lead polyamines potentiate antibiotic activity toward other ESKAPE pathogen species. *A. baumannii* (1643) or MRSA (USA300) cells were treated with the noted polyamine molecules at increasing concentrations, alongside levofloxacin **(A)** or chloramphenicol **(B)**. Shown is the fold potentiation of each antibiotic (EC_90_ values, blue bars) as the efflux pump inhibitor concentration was increased, and the concomitant decline in antibiotic MIC (EC_90_ values, red bars). Error bars are shown ± SEM.

To confirm that the activity of our lead agents was not merely confined to tetracycline, we next explored the potentiation of an unrelated antibiotic efflux substrate, chloramphenicol (**Figure 3B** and Supplementary Figure S2B). Each of the polyamine agents again displayed an increase in the potentiation of chloramphenicol EC_90_ in a dose responsive manner. Agent **266** displayed the highest potentiation values, although all compounds performed in a markedly similar, and effective manner. To determine if *P. aeruginosa* was the only organism targeted by our polyamine lead molecules, we next performed similar experiments with two other leading, and unrelated, ESKAPE pathogens: *A. baumannii* and Methicillin Resistant *S. aureus* (MRSA). It should be noted that in these studies, we only used a single compound for each, for proof of principle, due to limited available material. For the former organism we used lead molecule **271** and the antibiotic efflux substrate levofloxacin, again revealing a dose response curve of antibiotic potentiation as seen with *P. aeruginosa* (**Figure 4A**). When MRSA was tested in a similar manner using the substrate chloramphenicol and lead **247**, we also noted marked potentiation of antibiotic activity in a dose responsive manner (**Figure 4B**). As such, it would appear that our polyamines are capable of potentiating the activity of multiple antibiotic substrates, from a variety of different classes, and toward both Gram-negative and Gram-positive pathogens.

### Polyamines Function via the Inhibition of Bacterial Efflux Mechanisms

**FIGURE 5:**
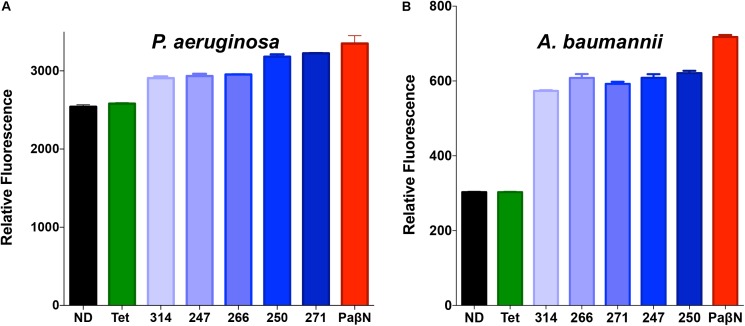
Polyamine molecules have broad spectrum efflux pump inhibitor activity. *P. aeruginosa* (1419) **(A)** or *A baumannii* (1643) **(B)** cells were treated with a sub-lethal concentration of ethidium bromide (25 μM) in combination with tetracycline, the known efflux inhibitor PAβN, lead polyamine agents at 25 μg mL^-1^, or vehicle (10% DMF) (ND). Graphs demonstrate fluorescence after 90-min exposure displayed as relative fluorescent units. Error bars are shown ± SEM.

Following these promising results, we sought to validate our findings using more direct means. Accordingly, the polyamines were assessed using an ethidium bromide fluorescence assay that has been widely used to identify efflux inhibitors (Renau et al., 1999; Lomovskaya et al., 2001; Webber et al., 2013; Blanchard et al., 2014; Vasudevan et al., 2014). This assay is a well documented variation on ethidium bromide accumulation assays, as it allows for a pre-incubation step to facilitate accumulation, before compound treatment to measure efflux (Raherison et al., 2002; Paixao et al., 2009). Fluorescence of ethidium bromide occurs during intercalation with DNA; thus, active efflux mechanisms decrease such fluorescence by extruding ethidium bromide before it can interact with its target. Thus, disruption of efflux pump activity leads to the accumulation of intracellular ethidium bromide and a subsequent increase in fluorescence over time compared to efflux proficient cells. Importantly, when we treated *P. aeruginosa* with lead polyamines, followed by a sub-lethal concentration of ethidium bromide, we observed an increase in fluorescence (**Figure 5A**) compared to no drug controls; indicating inhibition of efflux systems. To determine if these effects are solely limited to *P. aeruginosa*, we next tested other Gram-negative pathogens. When these assays were repeated with *A. baumannii*, we observed similar results (**Figure 5B**), indicating the broad-spectrum nature of these agents. Furthermore, when we assayed the gram-positive pathogen *S. aureus*, we again derived similar findings (Supplementary Figure S3A).

**FIGURE 6:**
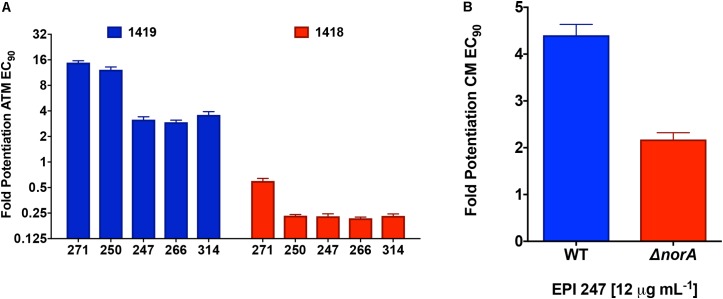
Confirming efflux pump inhibition by polyamines using efflux deficient strains. **(A)** A *P. aeruginosa* proficient (1419) and deficient (1418) strain were subjected to combination testing of the polyamines at 12.5 μg mL^-1^ and ½ X MIC of aztreonam. Fold potentiation was calculated by dividing the CFU mL^-1^ for aztreonam only by the CFU mL^-1^ of the combined treatment groups. **(B)** Lead polyamine **247** was assessed for its ability to potentiate the activity of chloramphenicol toward *S. aureus* strain United States 300 and its *norA* mutant. Shown is the potentiated chloramphenicol EC_90_ calculated as described in **A**. Error bars are shown ± SEM.

To provide further direct evidence of efflux inhibition by our lead compounds, we next tested their activity using strains with varying efflux potential. As such, alongside our test *P. aeruginosa* strain (1419) which has strong efflux capacity, we also tested an efflux impaired strain of this same organism, 1418. The efflux capacity of these two strains was determined using an ethidium bromide agar assay that has been previously documented as a measure of efflux capacity for bacterial strains (Supplementary Figure S4; Martins et al., 2013). Here, we tested all 5 leads against the two strains and the clinically relevant efflux antibiotic substrate aztreonam (**Figure 6A**). Upon analysis we found that all of our lead molecules were capable of effectively potentiating the activity of aztreonam toward our test strain 1419, however no such inhibition was seen for isolate 1418. Interestingly, we observed strong activity correlation with these studies, as our two best potentiators of tetracycline (**271** and **250**) were also the best potentiators of aztreonam.

To ensure that these findings were not specific only to *P. aeruginosa*, we next tested one of our lead polyamines, **247** (only 1 used due to limited available compound), against an efflux deficient strain of MRSA. For this we used a mutant of the major efflux pump NorA in the United States 300 background. When tested, we observed that whilst our lead polyamine was able to strongly potentiate chloramphenicol activity in the wild-type strain, there was little potentiation observed in the *norA* knockout (**Figure 6B**). The small amount of potentiation seen in the mutant strain is likely a result of the fact that, although NorA is indeed the major efflux pump in *S. aureus*, others also exist in this organism (Costa et al., 2013). Importantly, the MRSA strain used in these studies was different to those for our ethidium bromide analysis (USA 300 vs. United States 100), presenting further evidence that our molecules are not strain (or species) specific in their effects. Collectively, these data suggest that our polyamine molecules are not only effective inhibitors of bacterial efflux mechanisms, but that these effects appear to be broad-spectrum in range.

### Polyamines Act Competitively With PAβN to Potentiate the Tetracycline Effective Concentration

To further confirm the efflux pump inhibitor activity of polyamine agents, we performed a checkerboard assessment to determine the relationship between our front runner molecules and the control compound PAβN. If our polyamine agents inhibit a target other than efflux pumps, then combination treatment would produce a synergistic action. However, if the agents are both inhibiting efflux pumps, the result of combination treatment would be antagonistic (Auerbach et al., 2010; Goodman et al., 2011). Upon analysis we observed a clear competitive interaction between PAβN and polyamine agents in the presence of tetracycline (Supplementary Figure S5). This further confirms that the polyamine agents are inhibiting efflux pumps of bacterial species.

### Polyamine Molecules Do Not Randomly Depolarize Prokaryotic or Eukaryotic Membranes

**FIGURE 7:**
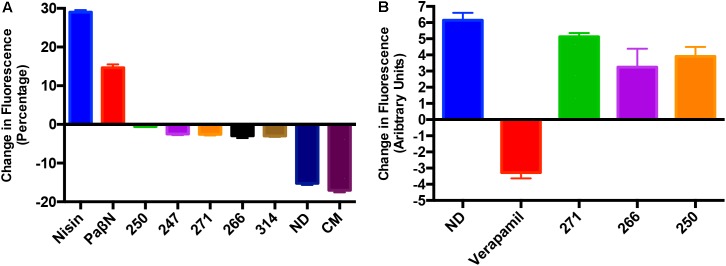
Polyamines do not destabilize prokaryotic or eukaryotic membranes. **(A)** Shown is the change in fluorescence of *P. aeruginosa* (1419) cells using a Disc3 dye assay. Data is presented as change in fluorescence before and after addition of leads compounds, PAβN, and nisin at 25 μg mL^-1^. **(B)** Calcium channel activity assays to assess inhibition of calcium channel pumps in HEK 293 cells after the addition of polyamines **250**, **266**, **271** or the positive control verapamil at 25 μg mL^-1^. Data is presented as change in fluorescence of cells before and after addition of the Fluo-4 dye. No drug (ND) and/or chloramphenicol (CM) were used as negative controls. Error bars are shown ± SEM.

A number of efflux pump inhibitors discovered to date have been shown to non-specifically inhibit efflux mechanisms through the non-specific depolarization of charge across bacterial membranes (Askoura et al., 2011; Webber et al., 2013). To determine if such effects were true of our polyamines, we assessed membrane depolarization using the molecular probe DiSC_3_. In cells with normal membrane polarity, the bacterial membrane will quench fluorescence of the DiSC_3_ dye. However, if the membrane is depolarized, the dye is released, and fluorescence increases over time. Our results reveal that the polyamines had minimal effect on bacterial membranes, as they allowed for stabilization of the quenched dye (**Figure 7A**). Whilst the positive control nisin and PAβN treated cell membranes displayed a strong increase in fluorescence, indicating membrane destabilization, cells treated with chloramphenicol or solvent only (10% DMF) decreased in florescence indicating a continued quenching of the membrane dye. Polyamines treated cells displayed essentially no change in fluorescence, revealing little to no depolarization when compared to the known efflux inhibitor PAβN. To ensure there is no depolarization of Gram-positive bacterial membranes we repeated this experiment with *S. aureus* and observed a lack of depolarization toward this species as well (Supplementary Figure S3B). As such, these results are considered promising given that other, unrelated polyamines have been shown to have the capacity to destabilize membranes by others (Katsu et al., 2002).

Another key consideration when developing efflux pump inhibitors is their impact on eukaryotic efflux systems, as many such molecules identified to date have non-specific effects on mammalian ion transport systems as well (Van Bambeke et al., 2006). This is of particular importance when testing polyamines, as other, unrelated molecules of this class have been shown to disrupt calcium release in bacterial cells as well as eukaryotic cells (Katsu et al., 2002). As such, we tested the effects of the polyamines in this regard against human embryonic kidney epithelial cells (Hek293T), alongside the known, and toxic efflux pump inhibitor, verapamil. In these studies, we determined that our polyamine efflux inhibitors mirrored no drug controls when assessed for their ability to interfere with eukaryotic calcium channel activity (**Figure 7B**). Specifically, lead compound treated cells exhibited increased fluorescence in the presence of the Fluoro-4 dye, whilst verapamil decreased fluorescence, representing the inhibition of calcium channel activity. Thus, it would appear that our polyamines are not only specific efflux pump inhibitors, but that their effects are selective for prokaryotic membrane pumps, over their eukaryotic counterparts.

### Lead Polyamine Efflux Pump Inhibitors Lack General Toxicity Toward Eukaryotic Cells

**FIGURE 8:**
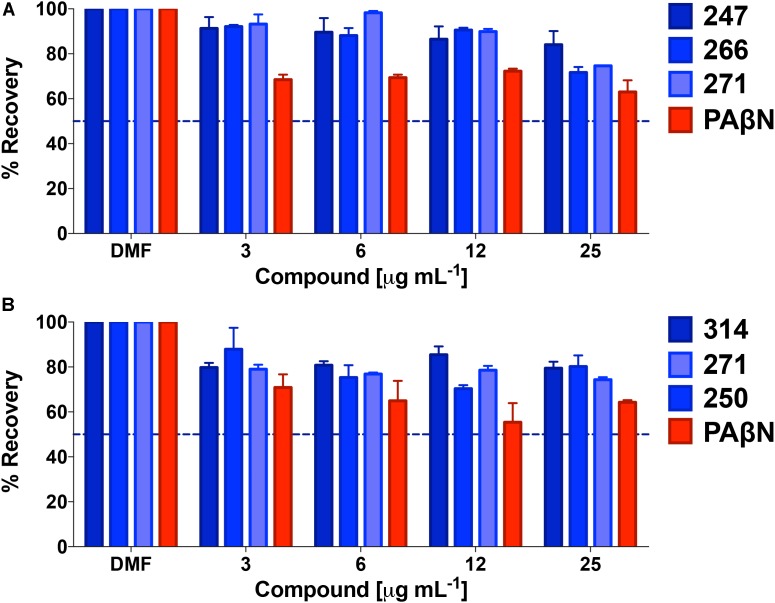
Lead polyamines lack general toxicity toward eukaryotic cells. Shown is the percent recovery of Hek293T cells **(A)** and HepG2 cells **(B)** when tested using a MTT cytotoxicity assay following treatment with polyamine leads. Conversion of MTT to formazan was assessed and converted to percent recovery using 0.01% Triton 100× as 100% death, and no treatment (DMF) as 100% survival. These controls were used to calculate percent recovery, and to determine LD_50_s (dotted line). Data is from at least three biological replicates, with error bars shown ± SEM.

Given the lack of effect of polyamines toward eukaryotic ion channels, we next assessed general cytotoxicity toward human cells. As such, polyamine lead compounds were tested against both HepG2 and Hek293T cell lines using MTT assays (**Figure 8**). In so doing, we determined that front runners **247, 266,** and **271** had extremely low toxicity toward Hek293T cells. Specifically, when treated with 25 μg mL^-1^ of these compounds, cells displayed 84, 72, and 75% recovery compared to solvent only controls, whilst the known efflux pump inhibitor PAβN returned only 63% cell viability. In support of this, HepG2 cell recovery after treatment with **250, 314, or 271** generated similar results; even at the highest concentration tested (again 25 μg mL^-1^) we observed 80, 77, and 74% cell viability. In comparison, the known efflux inhibitor PAβN tested at the same concentration allowed for 68% recovery of HepG2 cells. The higher toxicity of PAβN was perhaps unsurprising considering that this agent has been shown to depolarize membranes at higher concentrations (Webber et al., 2013).

### Polyamine Efflux Pump Inhibitors Strongly Enhance the Bactericidal Activity of Tetracycline

**FIGURE 9:**
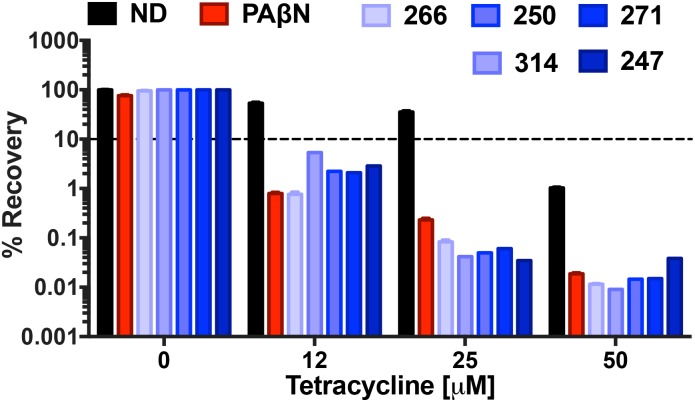
Polyamine efflux pump inhibitors strongly enhance the bactericidal activity of tetracycline. *P. aeruginosa* (1419) cells were treated with tetracycline at 0, 12, 25, and 50 μM in combination with no drug (ND), or **247**, **250**, **266**, **271**, **314** and PAβN at 25 μg mL^-1^. The dotted line on the graph denotes 90% bactericidal activity. Data is from at least three biological replicates, with error bars shown ± SEM.

We next set out to explore the impact of polyamines on the bactericidal effects of tetracycline. The rationale for this was that, although tetracycline is a bacteriostatic antibiotic, it is known to be bactericidal at high concentrations (Pankey and Sabath, 2004). Treatment with our polyamines alone at 25 μg mL^-1^ resulted in minimal impact to bacterial viability, with ≥95% of cells recovered for all compounds, in contrast to PAβN which returned only 76% viability at the same concentration. Tetracycline treatment alone at 12, 25, and 50 μM allowed for 53, 35, and 1% respective bacterial recovery. However, combination treatment with tetracycline and the MPC of all lead agents resulted in decreased bacterial viability. For example, combination treatment with 12 μM of tetracycline and polyamine **266** displayed the greatest decrease in bacterial viability, similar to the control PAβN. Specifically, the percent recovery decreased to 0.76 and 0.79% when treated with **266** or polyamine PaβN, respectively (**Figure 9**). Although not as impressive as **266** and PAβN, combination treatment with polyamines **247**, **250**, **271**, and **314** resulted in 2.9, 2.2, 2, and 5.3% recovery, respectively. Interestingly, we found that increasing tetracycline alone from 12 to 25 μM resulted in 17.8% less recovery, however combination treatment revealed a significant decrease in bacterial viability. In combination with 25 μM tetracycline, our polyamines appeared to outperform PAβN as they allowed for ≤0.08% recovery, whilst combination treatment with PAβN allowed for 0.2% recovery. Furthermore, 50 μM treatment with tetracycline decreased bacterial viability to 1% alone, however this was drastically decreased with combination efflux pump inhibitor treatment. Specifically, polyamines **250**, **266**, and **314** resulted in the greatest decrease in bacterial recovery, allowing for 0.01% recovery. This was marginally less recovery than that of the control PAβN and polyamine **271**, which allowed for 0.02% recovery. Polyamine **247** displayed the least decrease in viability with combination treatment, although it still decreased bacterial recovery to 0.04%. Given that bactericidal activity is often preferred to bacteriostatic effects, particularly for immunocompromised patients, these findings are considered encouraging.

### Polyamine Potentiation of Tetracycline Activity Reduces Biofilms by *P. aeruginosa*

**FIGURE 10:**
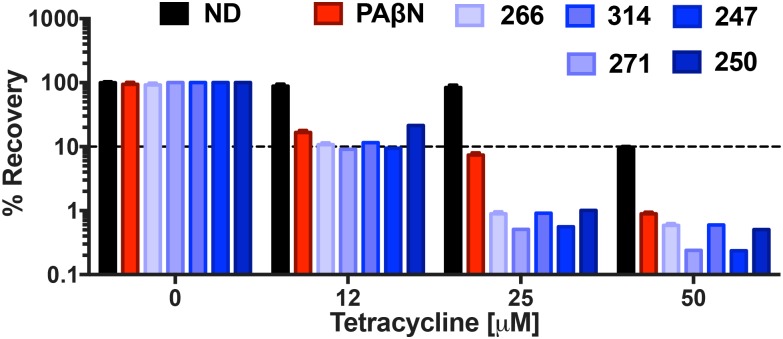
Polyamine potentiation of tetracycline activity limits biofilm recovery by *P. aeruginosa.* The lead polyamine agents were tested for their ability to impact viability of a pre-formed biofilm. *P. aeruginosa* (1419) cells were treated with tetracycline at 0, 12, 25, and 50 μM in combination with no drug (ND), or **247**, **250**, **266**, **271**, **314** and PAβN at 25 μg mL^-1^. The dotted line on the graph denotes 90% reduction in cell viability within biofilms. Data is from at least three biological replicates, with error bars shown ± SEM.

Biofilm formation is responsible for chronic, drug-resistant bacterial infections by a number of pathogens, and particularly *P. aeruginosa*. Considering the strong potentiation of tetracycline activity engendered by our lead agents, we next chose to determine if they had significant impact on the viability of cells within biofilms. Treatment with efflux pump inhibitors **247**, **250**, **266**, **271**, and **314** alone at 25 μg mL^-1^ (MPC), respectively, resulted in negligible impact on biofilm viability, with 99.99% of cells recovered for all compounds other than **266** which allowed for 92% recovery. Similarly, tetracycline treatment alone at 12 and 25 μM had little impact, allowing for 88 and 84% biofilm recovery, respectively (**Figure 10**); only at 50 μM were more pronounced effects observed, with only 9% cells recovered. Combination treatment with 12 μM tetracycline and the MPC of all lead agents resulted in a significant decrease in biofilm recovery, however, with viability of 9.1–11% observed for **271**, **247**, **266**, and **314**, respectively. Combination treatment with lead agent **250** resulted in a slightly higher biofilm recovery of 21%, however still improving tetracycline alone biofilm eradication by 67%. Increasing tetracycline concentration by itself from 12 to 25 μM only resulted in 4% more eradication, however in combination with our efflux pump inhibitors, recovery decreased to 0.5 and 0.6% for **271** and **247**, respectively. Similarly, treatment with agents **266**, **250**, and **314** resulted in 1% biofilm recovery. This biofilm eradication was particularly impressive when compared to the activity of the positive control PAβN (7% recovery at 25 μM tetracycline). Furthermore, at the highest tetracycline concentration (50 μM) combination treatment with PaβN produced a 1% biofilm recovery while agents **271** and **247** allowed for only 0.2% recovery. Treatment with agent **250**, **266**, and **314** resulted in similar recovery of 0.5, 0.6, and 0.6%, respectively. These results suggest a potential benefit of combination treatment with our polyamine molecules and known efflux antibiotics to reduce biofilms.

## Discussion

Antibiotic resistance is a developing crisis in clinical settings, with an increasing number of bacterial isolates discovered each year that are resistant to our therapeutic arsenal (Rossolini et al., 2014; Ventola, 2015). Efflux pumps are a major contributor to multi-drug resistance because they help circumvent the action of a broad range of substrates that includes multiple antibiotics classes (Askoura et al., 2011; Handzlik et al., 2013; Soto, 2013). This is compounded by the observation that most bacterial species utilize multiple efflux pumps with an overlapping range of substrates (Weinstein and Hooper, 2005; Van Bambeke et al., 2006; Sun et al., 2014). With this considered, the development of broadly active efflux pump inhibitors is considered desirable, so as to focus on the reclamation or reactivation of a wide swath of existing therapeutics (Masuda et al., 2000; Lomovskaya et al., 2001; Kumar and Varela, 2012; Blanchard et al., 2014).

The potentiating modeling utilized in this study allowed for the identification of polyamines that increased the effectiveness of tetracycline without displaying any toxic effects themselves. This highlights the importance of potentiation modeling for the identification of anti-resistance agents, as opposed to synergistic agents that display antimicrobial properties as well. Potentiation modeling is a more advantageous approach to identifying adjuvant agents because synergy assessment is reliant on the therapeutic agent having antibacterial activity (Hoel, 1987). Efflux pump inhibitors identified using synergistic activities, i.e., PaβN, have been unsuccessful due to off-target effects, causing bacterial growth inhibition (Renau et al., 1999; Lomovskaya et al., 2001; Webber et al., 2013). Importantly, in support of our approach, it has been shown that the concomitant treatment with an antibiotic and adjuvant agent that blocks the mechanism of resistance toward that antibiotic, but that has no antimicrobial properties itself, can lead to decreased resistance development (Olofsson and Cars, 2007; Worthington and Melander, 2013). Although a subset of polyamines was discovered during our screening with antimicrobial effect themselves, their structure activity relationship was taken into consideration during lead polyamine selection as discussed in detail below, and these molecules were eliminated from further consideration.

Efflux pump discovery began with the finding that efflux of host antimicrobial polyamines by the *S. aureus* Tet38 efflux pump facilitated skin colonization, and the ability to survive within an abscess environment (Truong-Bolduc et al., 2013). Beginning with the initial development of polyamine tetracycline derivatives to increase the effectiveness of tetracycline, (Nelson et al., 1993, 1994; Nelson and Levy, 1999), polyamine molecules have been shown to increase the therapeutic potential of common antibiotics (Vazquez-Laslop, 1997; Kwon and Lu, 2007; Rockey et al., 2013). Kwon and Lu (2007) revealed the endogenous polyamines spermidine and spermine, found within all living cells, when administered exogenously were effective at increasing the therapeutic potential of β-lactams toward Gram-negative organisms by blocking the outer membrane porin OprD. They also found these natural polyamines were shown to potentiate activity of chloramphenicol and β-lactams toward *Escherichia coli* and *S. aureus* (Kwon and Lu, 2007). Similarly, in *Bacillus subtilis*, the efflux pump Blt is dedicated to the extrusion of spermidine, however it has also been shown to opportunistically bind to other polyamine molecules (Vazquez-Laslop, 1997). Furthermore, in *Mycobacterium tuberculosis*, there is a significant increase in the effectiveness of fluoroquinolones administered alongside polyamine treatment (Rockey et al., 2013). Conversely, it has been shown that natural polyamines and PaβN have toxicity associated with their amine substituents, therefore care must be taken when developing therapeutics of this nature (Opperman et al., 2013; Pegg, 2013). Even though this toxicity is known, amine molecules continue to be pursued to target multiple disease states (Goldberg and Rajfer, 1982; Thomas and Thomas, 2001; Bogatcheva et al., 2006; de Jonge et al., 2015; Li et al., 2015a). Moreover, there are many therapeutics in use in clinics today that contain amines, such as aminoglycosides; thus, despite their potential for toxicity, approaches have been used successful to decrease these effects (Poulikakos and Falagas, 2013). Indeed, the FDA recently approved Zerbaxa (ceftolozane/tazobactam) for treatment of β-lactamase producing enteric species. Importantly, the ceftolozane component of this therapeutic combination contains multiple amine substituents (Fernandes and Martens, 2017). These studies collectively reveal the therapeutic potential of polyamine molecules despite the potential for compounds of this class to display unwanted side-effects.

The polyamine agents discovered in this study were successful in not only returning the effectiveness of tetracycline but also an unrelated antibiotic efflux substrate, chloramphenicol. Importantly, this finding reveals that our polyamines are not just allowing for the increased effectiveness of one, but multiple commercial antibiotics from a broad range of different classes. This finding suggests that the polyamines discovered in our study inhibit through direct competitive inhibition (Kourtesi, 2013). This mechanism of efflux inhibition capitalizes on the broad-spectrum binding affinity of efflux pumps by blocking the substrate binding extrusion protomer on the distal binding site (Nikaido and Pages, 2012; Opperman and Nguyen, 2015). Interestingly, tetracycline binds to the “groove” region of the binding pocket in the binding protomer, while chloramphenicol binds to the deeper “cave” region. Covalent binding to this “cave” region is the ultimate in efflux inhibition as it causes the binding pocket to collapse and become non-functional, therefore inhibiting multiple substrates from binding (Nikaido and Pages, 2012).

Notably, the direct measurement of ethidium bromide fluorescence revealed our polyamines inhibit the efflux pumps of Gram-negative species *P. aeruginosa* and *A. baumannii*, as well as the Gram-positive organism *S. aureus*. There are five families of efflux pumps: MFS, ABC, SMR, MATE, and RND. The first four are used by all bacterial species, whilst RND efflux pumps are largely used by Gram-negative species (Sun et al., 2014). The RND family is the main Gram-negative efflux system suggesting that, as our polyamine agents are active in both Gram-positive and -negative organisms, they are likely inhibiting more than one family of efflux pumps (although it is acknowledged that some Gram-positive organisms do harbor them). This can be attributed to the competitive inhibition nature of efflux pumps inhibitors that harnesses the broad substrate recognition of efflux pumps for more effective efflux inhibition in multiple organisms. For example, the efflux pump families of RND, ABC, SMR, and MFS all recognize substrates with polycationic properties (Kumar and Pooja Patial, 2016). Further to this, RND pumps found in *P. aeruginosa* and *E. coli* recognize and extrude tetracycline, while ABC and SMR pumps in *S. aureus* also expel this same antibiotic (Kumar and Pooja Patial, 2016). This would explain the activity of the previously identified efflux pump inhibitor baicalein, which is derived from the plant *Thymus vulgaris*, and has been found to potentiate tetracycline activity by blocking the MFS family TetK efflux pumps of *E. coli* and *S. aureus* (Wax et al., 2001; Fujita et al., 2005; Kumar and Pooja Patial, 2016). Moreover, our polyamine efflux pump inhibitors appear to be acting in a competitive manner with the positive control PAβN, potentially competing for the same substrate binding pocket (Webber et al., 2013; Venter et al., 2015). In fact, our polyamines resemble the known efflux pump inhibitor PAβN more so than the other well-known efflux pump inhibitor 1-(1-naphthylmethyl)-piperazine (NMP), which is shown not to potentiate antibiotic activity toward *P. aeruginosa* (Coban et al., 2009). This would explain the activity of the recent identification of a pyranopyridine inhibitor, MBX2319 that was designed based on NMP and found to have potent activity toward Enterobacteriaceae but little activity toward *P. aeruginosa* (Sjuts et al., 2016). However, a pyridopyrimidine scaffold discovered by Nakashima et al. (2013), with more similarities to PAβN was found to potentiate chloramphenicol and tetracycline and further revealed to bind the distal pocket of both *P. aeruginosa* MexAB and *E. coli* AcrB, whereas MBX2319 was specific to AcrB (Venter et al., 2015). However, even with the apparent binding differences observed in recent studies, MexAB of *P. aeruginosa* is still a close homolog of AcrB and has the same dependence on outer membrane stability as all RND efflux pumps do (Li et al., 2015b). Therefore, this dependence on membrane stability necessitates a rigorous analysis of membrane disruption for all agents (polyamine or otherwise) being developed as efflux pump inhibitors.

Importantly, we observed our polyamines did not have deleterious effects on bacterial cell membranes, as is seen for the known efflux pump inhibitor PAβN (Webber et al., 2013). Many efflux pump inhibitors discovered to date have been found to cause disruption of the bacterial cell membrane leading to their ineffectiveness as therapeutic agents (Misra and Bavro, 2009; Machado et al., 2017). Disruption of the bacterial cell membrane causes inhibition of efflux through a secondary effect of membrane depolarization, leading to inhibitory activity alone, and the common identification of false positive, non-specific efflux pump inhibitors (Cornwell et al., 1987; Webber et al., 2013; Li et al., 2015b). Considering the fact that polyamines have been shown to disrupt bacterial membranes it is, perhaps surprising that our polyamines do not disturb bacterial membranes, however the potentiation modeling used herein specifically sought to eliminate the selection of false positive efflux pump inhibitors. In our analysis we did identify polyamines, derived from our scaffold, with antimicrobial activity that was eliminated from study due to unwanted, off-target effects. Regarding our lead agents, at least at the maximum concentrations tested, we observed little to no membrane destabilization for MRSA and *P. aeruginosa*. We note, however, that other polyamine molecules do have the potential for this activity, thus these types of studies will be an important part of future development for our frontrunners. The identification of 37 polyamines that inhibited bacterial viability themselves, although less than ideal, lead to knowledge of a structure activity relationship for efflux pump specificity. Those polyamines found to inhibit bacterial viability themselves may be disturbing the cellular membrane based on the position of positive charges within their structure. Our analysis revealed 24% of the polyamines (26 out of 103) with R1 and R2 both defined by *S*-methylbenzene lead to antibacterial activity, while only 7% (4 out of 60) that have *S*-methylbenzene at R2 and R3, and 12% (7 out of 60) that have *S*-methylbenzene at R1 and R3, had antibacterial activity. With this knowledge, we focused our attention on compounds that had no antibacterial activity themselves to avoid selecting membrane depolarizing agents. In the future development of our molecules, iterative structure activity relationship studies will ensure bias away from such unwanted activity, and focus on harnessing only the positive, efflux pump inhibiting effects observed for our lead molecules.

To increase our confidence in the specificity of the polyamines discovered herein, we demonstrate that they have limited toxicity toward two human cell lines and no inhibitory effects on the eukaryotic Ca^2+^ channel activity of human kidney cells. To date, many efflux pump inhibitors (such as verapamil) have been shown to have non-specific inhibition of eukaryotic transporters, and therefore create unwanted side effects as therapeutic agents (Lomovskaya et al., 2001; Ughachukwu and Unekwe, 2012; Amin, 2013; Lebeaux et al., 2013). Verapamil is a non-specific inhibitor of calcium channels, found to also inhibit the function of P-glycoprotein ABC transporters in mammalian cells (Andersen et al., 2006). This inhibitor was found to have *in vitro* function as a bacterial efflux inhibitor, however due to its general toxicity, the use of this compound is limited to angina, hypertension, and cardiac arrhythmia (Andersen et al., 2006; Gupta et al., 2013). This assessment is extremely important for our polyamines as calcium release has also been observed from polyamines on bacterial cells (Katsu et al., 2002). With limited toxicity and secondary effects, the polyamine efflux pump inhibitors discovered in our study appear to have more favorable characteristics than others previously discovered, suggesting promising potential as adjuvant agents.

Taken together, the polyamines discovered in this study have potential as therapeutic adjuvants to rescue the effects of multiple antibiotics toward both Gram-positive and Gram-negative species. They appear to be acting in a specific manner and have none of the undesirable membrane targeting characteristics of previously developed efflux pump inhibitors. As such, we suggest that the polyamines developed herein are a promising scaffold for further development of anti-resistance agents to help alleviate the burden of multi-drug resistant bacterial pathogens.

## Author Contributions

Project was designed by RF, MG, and LS. Experiments were performed by RF, JA, GD, and KA. Data was analyzed by RF, RS, GW, RH, MG, and LS. Manuscript was written by RF, JA, RH, MG, and LS. Funding was provided by RH and LS.

## Conflict of Interest Statement

The authors declare that the research was conducted in the absence of any commercial or financial relationships that could be construed as a potential conflict of interest.

## References

[B1] Adabi M. Talebi-Taher M. Arbabi L. Afshar M. Fathizadeh S. Minaeian S. (2015). Spread of efflux pump overexpressing-mediated fluoroquinolone resistance and multidrug resistance in *Pseudomonas aeruginosa* by using an efflux pump Inhibitor. *Infect. Chemother.* 47 98–104. 10.3947/ic.2015.47.2.98 26157587 PMC4495281

[B2] Amin M. L. (2013). P-glycoprotein inhibition for optimal drug delivery. *Drug Target Insights* 7 27–34. 10.4137/DTI.S12519 24023511 PMC3762612

[B3] Andersen C. L. Holland I. B. Jacq A. (2006). Verapamil, a Ca2+ channel inhibitor acts as a local anesthetic and induces the sigma E dependent extra-cytoplasmic stress response in *E. coli*. *Biochim. Biophys. Acta* 1758 1587–1595. 10.1016/j.bbamem.2006.05.022 16836975

[B4] Askoura M. Mattawa W. Abujamel T. Taher I. (2011). Efflux pump inhibitors (EPIs) as new antimicrobial agents against *Pseudomonas aeruginosa*. *Libyan J. Med.* 6:5870. 10.3402/ljm.v6i0.5870 21594004 PMC3096568

[B5] Auerbach T. Mermershtain I. Davidovich C. Bashan A. Belousoff M. Wekselman I. (2010). The structure of ribosome-lankacidin complex reveals ribosomal sites for synergistic antibiotics. *Proc. Natl. Acad. Sci. U.S.A.* 107 1983–1988. 10.1073/pnas.0914100107 20080686 PMC2804743

[B6] Blanchard C. Barnett P. Perlmutter J. Dunman P. M. (2014). Identification of *Acinetobacter baumannii* serum-associated antibiotic efflux pump inhibitors. *Antimicrob. Agents Chemother.* 58 6360–6370. 10.1128/AAC.03535-14 25114126 PMC4249429

[B7] Bogatcheva E. Hanrahan C. Nikonenko B. Samala R. Chen P. Gearhart J. (2006). Identification of new diamine scaffolds with activity against *Mycobacterium tuberculosis*. *J. Med. Chem.* 49 3045–3048. 10.1021/jm050948+ 16722620 PMC4869334

[B8] Boucher H. W. Talbot G. H. Bradley J. S. Edwards J. E. Gilbert D. Rice L. B. (2009). Bad bugs, no drugs: no ESKAPE! An update from the Infectious Diseases Society of America. *Clin. Infect. Dis.* 48 1–12. 10.1086/595011 19035777

[B9] Chamberland S. Hecker S. J. Lee V. J. Trias J. (1996). WO9633285.

[B10] Chamberland S. Ishida H. Lee V. J. Leger R. Nakayama K. Ohta T. (2000). WO0001714.

[B11] Chamberland S. Lee M. Lee V. J. Leger R. Renau T. She M. W. (1999). WO9937667.

[B12] Chamberland S. Lee M. Leger R. Lee V. J. Renau T. Zhang J. Z. (2001). US6245746.

[B13] Chevalier D. P. Touboul D. P. Zipes D. P. Wellens H. J. J. (1999). Calcium-channel blockers and cardiac arrest response. *Circulation* 100:e140 10.1161/01.CIR.100.25.e140 10604907

[B14] ClinicalTrials.gov (2014). *A Service of the US National Institutes of Health.* NCT02092506 Available at: www.clinicaltrials.gov

[B15] Coban A. Y. Bayram Z. Sezgin F. M. Durupinar B. (2009). Effect of efflux pump inhibitor 1-(1-naphthylmethyl)-piperazine to MIC values of ciprofloxacin in ciprofloxacin resistant gram-negative bacteria. *Mikrobiyol. Bul.* 43 457–461. 19795621

[B16] Cornwell M. M. Pastan I. Gottesman M. M. (1987). “Certain calcium channel blockers bind specifically to multidrug-resistant human KB carcinoma membrane vesicles and inhibit drug binding to P-glycoprotein. *J. Biol. Chem.* 262 2166–2170. 2434476

[B17] Costa S. S. Viveiros M. Amaral L. Couto I. (2013). Multidrug efflux pumps in *Staphylococcus aureus*: an update. *Open Microbiol. J.* 7 59–71. 10.2174/1874285801307010059 23569469 PMC3617543

[B18] de Jonge B. L. Kutschke A. Newman J. V. Rooney M. T. Yang W. Cederberg C. (2015). Pyridodiazepine amines are selective therapeutic agents for *Helicobacter pylori* by suppressing growth through inhibition of glutamate racemase but are predicted to require continuous elevated levels in plasma to achieve clinical efficacy. *Antimicrob. Agents Chemother.* 59 2337–2342. 10.1128/AAC.04410-14 25645840 PMC4356811

[B19] De Souza N. Patel M. V. Gupte S. V. Upad-Hyay D. J. Shukla M. C. Chaturvedi N. C. (2002). WO0209758.

[B20] Falagas M. E. Bliziotis I. A. (2007). Pandrug-resistant Gram-negative bacteria: the dawn of the post-antibiotic era? *Int. J. Antimicrob. Agents* 29 630–636. 10.1016/j.ijantimicag.2006.12.012 17306965

[B21] Fernandes P. Martens E. (2017). Antibiotics in late clinical development. *Biochem. Pharmacol.* 133 152–163. 10.1016/j.bcp.2016.09.025 27687641

[B22] Fleeman R. LaVoi T. M. Santos R. G. Morales A. Nefzi A. Welmaker G. S. (2015). Combinatorial libraries as a tool for the discovery of novel, broad-spectrum antibacterial agents targeting the ESKAPE pathogens. *J. Med. Chem.* 58 3340–3355. 10.1021/jm501628s 25780985 PMC8982266

[B23] Fujita M. Shiota S. Kuroda T. Hatano T. Yoshida T. Mizushima T. (2005). Remarkable synergies between baicalein and tetracycline, and baicalein and beta-lactams against methicillin-resistant *Staphylococcus aureus*. *Microbiol. Immunol.* 49 391–396. 10.1111/j.1348-0421.2005.tb03732.x 15840965

[B24] Goldberg L. I. Rajfer S. I. (1982). Sympathomimetic amines: potential clinical applications in ischemic heart disease. *Am. Heart. J* 103(4 Pt 2), 724–729. 10.1016/0002-8703(82)90479-3 6278912

[B25] Goodman L. S. Brunton L. L. Chabner B. Knollmann B. R. C. (2011). *Goodman & Gilman’s Pharmacological Basis of Therapeutics.* New York, NY: McGraw-Hill.

[B26] Gupta S. Tyagi S. Almeida D. V. Maiga M. C. Ammerman N. C. Bishai W. R. (2013). Acceleration of tuberculosis treatment by adjunctive therapy with verapamil as an efflux inhibitor. *Am. J. Respir. Crit. Care Med.* 188 600–607. 10.1164/rccm.201304-0650OC 23805786 PMC3827702

[B27] Handzlik J. Matys A. Kieć-Kononowicz K. (2013). Recent advances in multi-drug resistance (MDR) efflux pump inhibitors of gram-positive bacteria *S. aureus*. *Antibiotics* 2 28–45. 10.3390/antibiotics2010028 27029290 PMC4790296

[B28] Hoel D. G. (1987). “Statistical aspects of chemical mixtures,” in *Methods for Assessing the Effects of Mixtures of Chemicals*, eds Vouk V. B. Butler G. C. Upton A. C. Parke D. V. Asher S. C. (New York, NY: Wiley).

[B29] Houghten R. A. Pinilla C. Appel J. R. Blondelle S. E. Dooley C. T. Eichler J. (1999). Mixture-based synthetic combinatorial libraries. *J. Med. Chem.* 42 3743–3778. 10.1021/jm990174v 10508425

[B30] Katsu T. Nakagawa H. Yasuda K. (2002). Interaction between polyamines and bacterial outer membranes as investigated with ion-selective electrodes. *Antimicrob. Agents Chemother.* 46 1073–1079. 10.1128/AAC.46.4.1073-1079.2002 11897592 PMC127117

[B31] Kourtesi C. (2013). Microbial efflux systems and inhibitors: approaches to drug discovery and the challenge of clinical implementation. *Open Microbiol. J.* 7 34–52. 10.2174/1874285801307010034 23569468 PMC3617545

[B32] Kumar R. Pooja Patial S. J. (2016). A review on efflux pump inhibitors of gram-positive and gram-negative bacteria from plant sources. *Int. J. Curr. Microbiol. Appl. Sci.* 5 837–855. 10.20546/ijcmas.2016.506.092

[B33] Kumar S. Varela M. F. (2012). Biochemistry of bacterial multidrug efflux pumps. *Int. Jo. Mol. Sci.* 13 4484–4495. 10.3390/ijms13044484 22605991 PMC3344227

[B34] Kwon D. H. Lu C. D. (2007). Polyamine effects on antibiotic susceptibility in bacteria. *Antimicrob. Agents Chemother.* 51 2070–2077. 10.1128/AAC.01472-06 17438056 PMC1891406

[B35] Lebeaux D. Chauhan A. Rendueles O. Beloin C. (2013). from in vitro to in vivo models of bacterial biofilm-related infections. *Pathogens* 2 288–356. 10.3390/pathogens2020288 25437038 PMC4235718

[B36] Lemaire M. Moreau N. Fournier Dit C. J. Chabert J. Marquez B. Marquet B. (2006). WO2006018544.

[B37] Levy S. B. (1998). Reducing tetracycline resistance in living cells. U.S. Patent No US5811412 Washington, DC: U.S. Patent and Trademark Office.

[B38] Li J.-L. Zhao W. Zhou C. Zhang X. Y. Li H. M. Tang Y. L. (2015a). Comparison of carbon-sulfur and carbon-amine bond in therapeutic drug: 4β-S-aromatic heterocyclic podophyllum derivatives display antitumor activity. *Sci. Rep.* 5:14814. 10.1038/srep14814 26443888 PMC4595834

[B39] Li X. Z. Plesiat P. Nikaido H. (2015b). The challenge of efflux-mediated antibiotic resistance in Gram-negative bacteria. *Clin. Microbiol. Rev.* 28 k337–418. 10.1128/CMR.00117-14 25788514 PMC4402952

[B40] Lister P. D. Wolter D. J. Hanson N. D. (2009). Antibacterial-resistant *Pseudomonas aeruginosa*: clinical impact andex regulation of chromosomally encoded resistance mechanisms. *Clin. Microbiol. Rev.* 22 582–610. 10.1128/CMR.00040-09 19822890 PMC2772362

[B41] Lomovskaya O. Warren M. S. Lee A. Galazzo J. Fronko R. Lee M. (2001). Identification and characterization of inhibitors of multidrug resistance efflux pumps in *Pseudomonas aeruginosa*: novel agents for combination therapy. *Antimicrob. Agents Chemother.* 45 105–116. 10.1128/AAC.45.1.105-116.2001 11120952 PMC90247

[B42] Machado D. Fernandes L. Costa S. S. Cannalire R. Manfroni G. Tabarrini O. (2017). Mode of action of the 2-phenylquinoline efflux inhibitor PQQ4R against *Escherichia coli*. *PeerJ* 5:e3168. 10.7717/peerj.3168 28516003 PMC5433425

[B43] Markham P. N. Mulhearn D. C. Neyfakh A. A. Crich D. Jaber M. R. Johnson M. E. (2000). US99/28732.

[B44] Martins M. McCusker M. P. Viveiros M. Couto I. Fanning S. Pages J. M. (2013). A Simple method for assessment of MDR bacteria for over-expressed efflux pumps. *Open Microbiol. J.* 7 72–82. 10.2174/1874285801307010072 23589748 PMC3624690

[B45] Masuda N. Sakagawa E. Ohya S. Gotoh N. Tsujimoto H. Nishino T. (2000). Substrate specificities of MexAB-OprM, MexCD-OprJ, and MexXY-oprM efflux pumps in *Pseudomonas aeruginosa*. *Antimicrob. Agents Chemother.* 44 3322–3327. 10.1128/AAC.44.12.3322-3327.2000 11083635 PMC90200

[B46] McMurry L. Petrucci R. E. Jr. Levy S. B. (1980). Active efflux of tetracycline encoded by four genetically different tetracycline resistance determinants in *Escherichia coli*. *Proc. Natl. Acad. Sci. U.S.A.* 77 3974–3977. 10.1073/pnas.77.7.3974 7001450 PMC349750

[B47] Misra R. Bavro V. N. (2009). Assembly and transport mechanism of tripartite drug efflux systems. *Biochim. Biophys. Acta* 1794 817–825. 10.1016/j.bbapap.2009.02.017 19289182 PMC2671569

[B48] Nakashima R. Sakurai K. Yamasaki S. Hayashi K. Nagata C. Hoshino K. (2013). Structural basis for the inhibition of bacterial multidrug exporters. *Nature* 500 102–106. 10.1038/nature12300 23812586

[B49] Nakayama K. Ohtsuka M. Haruko K. Ryo O. Kazuki H. Watkins W. (2002). WO02087589.

[B50] Nelson M. L. Alekhsun M. N. (2004). WO2004/062674.

[B51] Nelson M. L. Levy S. B. (1999). Reversal of tetracycline resistance mediated by different bacterial tetracycline resistance determinants by an inhibitor of the Tet(B) antiport protein. *Antimicrob. Agents Chemother.* 43 1719–1724. 10390229 10.1128/aac.43.7.1719 PMC89350

[B52] Nelson M. L. Park B. H. Andrews J. S. Georgian V. A. Thomas R. C. Levy S. B. (1993). Inhibition of the tetracycline efflux antiport protein by 13-thio-substituted 5-hydroxy-6-deoxytetracyclines. *J. Med. Chem.* 36 370–377. 10.1021/jm00055a008 8426364

[B53] Nelson M. L. Park B. H. Levy S. B. (1994). Molecular requirements for the inhibition of the tetracycline antiport protein and the effect of potent inhibitors on the growth of tetracycline-resistant bacteria. *J. Med. Chem.* 37 1355–1361. 10.1021/jm00035a016 8176712

[B54] Neyrolles O. Machado D. Pires D. Perdigão J. Couto I. Portugal I. (2016). Ion Channel blockers as antimicrobial agents, efflux inhibitors, and enhancers of macrophage killing activity against drug resistant *Mycobacterium tuberculosis*. *PLoS One* 11:e0149326. 10.1371/journal.pone.0149326 26919135 PMC4769142

[B55] Nikaido H. Pages J. M. (2012). Broad-specificity efflux pumps and their role in multidrug resistance of Gram-negative bacteria. *FEMS Microbiol. Rev.* 36 340–363. 10.1111/j.1574-6976.2011.00290.x 21707670 PMC3546547

[B56] Olofsson S. K. Cars O. (2007). Optimizing drug exposure to minimize selection of antibiotic resistance. *Clin. Infect. Dis.* 45(Suppl. 2), S129–S136. 10.1086/519256 17683017

[B57] Opperman T. J. Kwasny S. M. Kim H. S. Nguyen S. T. Houseweart C. D’Souza S. (2013). Characterization of a novel pyranopyridine inhibitor of the AcrAB efflux pump of *Escherichia coli*. *Antimicrob. Agents Chemother.* 58 722–733. 10.1128/AAC.01866-13 24247144 PMC3910843

[B58] Opperman T. J. Nguyen S. T. (2015). Recent advances toward a molecular mechanism of efflux pump inhibition. *Front. Microbiol.* 6:421 10.3389/fmicb.2015.00421 PMC4419859 25999939

[B59] Pages J. M. Mallea M. Chevalier J. Barbe J. Abdallah M. Kayirere M. G. (2003). FR2839647.

[B60] Paixao L. Rodrigues L. Couto I. Martins M. Fernandes P. de Carvalho C. C. (2009). Fluorometric determination of ethidium bromide efflux kinetics in *Escherichia coli*. *J. Biol. Eng.* 3:18. 10.1186/1754-1611-3-18 19835592 PMC2774284

[B61] Palmer G. C. Whiteley M. (2015). Metabolism and pathogenicity of *Pseudomonas aeruginosa* infections in the lungs of individuals with cystic fibrosis. *Microbiol. Spectr.* 3 185–213. 26350318 10.1128/microbiolspec.MBP-0003-2014

[B62] Pankey G. A. Sabath L. D. (2004). Clinical relevance of bacteriostatic versus bactericidal mechanisms of action in the treatment of gram-positive bacterial infections. *Clin. Infect. Dis* 38 864–870. 10.1086/381972 14999632

[B63] Pegg A. E. (2013). Toxicity of polyamines and their metabolic products. *Chem. Res. Toxicol.* 26 1782–1800. 10.1021/tx400316s 24224555

[B64] Poulikakos P. Falagas M. E. (2013). Aminoglycoside therapy in infectious diseases. *Exp. Opin. Pharmacother.* 14 1585–1597. 10.1517/14656566.2013.806486 23746121

[B65] Raherison S. Gonzalez P. Renaudin H. Charron A. Bebear C. Bebear C. M. (2002). Evidence of active efflux in resistance to ciprofloxacin and to ethidium bromide by *Mycoplasma hominis*. *Antimicrob. Agents Chemother.* 46 672–679. 10.1128/AAC.46.3.672-679.2002 11850247 PMC127495

[B66] Reardon S. (2014). WHO warns against ‘post-antibiotic’ era. *Nature.* 10.1038/nature.2014.15135

[B67] Reilley K. J. Giulianotti M. Dooley C. T. Nefzi A. McLaughlin J. P. Houghten R. A. (2010). Identification of two novel, potent, low-liability antinociceptive compounds from the direct in vivo screening of a large mixture-based combinatorial library. *AAPS J.* 12 318–329. 10.1208/s12248-010-9191-3 20422341 PMC2895443

[B68] Renau T. E. Léger R. Flamme E. M. Sangalang J. She M. W. Yen R. (1999). Inhibitors of efflux pumps in *Pseudomonas aeruginosa* potentiate the activity of the fluoroquinolone antibacterial levofloxacin. *J. Med. Chem.* 42 4928–4931. 10.1021/jm9904598 10585202

[B69] Rockey D. Sarathy J. P. Lee E. Dartois V. (2013). Polyamines inhibit porin-mediated fluoroquinolone uptake in mycobacteria. *PLoS One* 8:e65806. 10.1371/journal.pone.0065806 23755283 PMC3670895

[B70] Rossolini G. M. Arena F. Pecile P. Pollini S. (2014). Update on the antibiotic resistance crisis. *Curr. Opin. Pharmacol.* 18 56–60. 10.1016/j.coph.2014.09.006 25254623

[B71] Sandhaus S. Annamalai T. Welmaker G. Houghten R. A. Paz C. Garcia P. K. (2016). Small-molecule inhibitors targeting topoisomerase I as novel antituberculosis agents. *Antimicrob. Agents Chemother.* 60 4028–4036. 10.1128/AAC.00288-16 27114277 PMC4914652

[B72] Santos R. G. Appel J. R. Giulianotti M. A. Edwards B. S. Sklar L. A. Houghten R. A. (2013). The mathematics of a successful deconvolution: a quantitative assessment of mixture-based combinatorial libraries screened against two formyl peptide receptors. *Molecules* 18 6408–6424. 10.3390/molecules18066408 23722730 PMC4106117

[B73] Sjuts H. Vargiu A. V. Kwasny S. M. Nguyen S. T. Kim H.-S. Ding X. (2016). Molecular basis for inhibition of AcrB multidrug efflux pump by novel and powerful pyranopyridine derivatives. *Proc. Natl. Acad. Sci. U.S.A.* 113 3509–3514. 10.1073/pnas.1602472113 26976576 PMC4822567

[B74] Soto S. M. (2013). Role of efflux pumps in the antibiotic resistance of bacteria embedded in a biofilm. *Virulence* 4 223–229. 10.4161/viru.23724 23380871 PMC3711980

[B75] Spellberg B. Guidos R. Gilbert D. Bradley J. Boucher H. W. Scheld W. M. (2008). The epidemic of antibiotic-resistant infections: a call to action for the medical community from the infectious diseases society of America. *Clin. Infect. Dis.* 46 155–164. 10.1086/524891 18171244

[B76] Sun J. Deng Z. Yan A. (2014). Bacterial multidrug efflux pumps: mechanisms, physiology and pharmacological exploitations. *Biochem. Biophys. Res. Commun.* 453 254–267. 10.1016/j.bbrc.2014.05.090 24878531

[B77] Thomas T. Thomas T. J. (2001). Polyamines in cell growth and cell death: molecular mechanisms and therapeutic applications. *Cell. Mol. Life Sci.* 58 244–258. 10.1007/PL00000852 11289306 PMC11146526

[B78] Tommasi R. Brown D. G. Walkup G. K. Manchester J. I. Miller A. A. (2015). ESKAPEing the labyrinth of antibacterial discovery. *Nat. Rev. Drug Discov.* 14 529–542. 10.1038/nrd4572 26139286

[B79] Truong-Bolduc Q. C. Villet R. A. Estabrooks Z. A. Hooper D. C. (2013). Native efflux pumps contribute resistance to antimicrobials of skin and the ability of *Staphylococcus aureus* to colonize skin. *J. Infect. Dis.* 209 1485–1493. 10.1093/infdis/jit660 24280365 PMC3982850

[B80] Ughachukwu P. O. Unekwe P. C. (2012). Efflux pump-mediated resistance in chemotherapy. *Ann. Med. Health Sci. Res.* 2 191–198. 10.4103/2141-9248.105671 23439914 PMC3573517

[B81] Van Bambeke F. Pages J. M. Lee V. J. (2006). Inhibitors of bacterial efflux pumps as adjuvants in antibiotic treatments and diagnostic tools for detection of resistance by efflux. *Recent Pat. Antiinfect. Drug Discov.* 1 157–175. 10.2174/157489106777452692 18221142

[B82] Van Horn K. S. Burda W. N. Fleeman R. Shaw L. N. Manetsch R. (2014). Antibacterial activity of a aeries ofN2,N4-disubstituted quinazoline-2,4-diamines. *J. Med. Chem.* 57 3075–3093. 10.1021/jm500039e 24625106

[B83] Vasudevan A. Dineshkumar K. Mohanalakshmi N. Velmurugan D. Hopper W. (2014). Identification of inhibitors for Rnd efflux pump of *Pseudomonas aeruginosa* using structure-based pharmacophore modeling approach. *Int. J. Pharm. Pharmaceut. Sci.* 6 84–89. 27450181

[B84] Vazquez-Laslop N. (1997). Efflux of the natural polyamine spermidine facilitated by the *Bacillus subtilis* multidrug transporter Blt. *J. Biol. Chem.* 272 8864–8866. 10.1074/jbc.272.14.8864 9083003

[B85] Venter H. Mowla R. Ohene-Agyei T. Ma S. (2015). RND-type drug efflux pumps from Gram-negative bacteria: molecular mechanism and inhibition. *Front. Microbiol.* 6:377 10.3389/fmicb.2015.00377 PMC4412071 25972857

[B86] Ventola C. L. (2015). The antibiotic resistance crisis: part 1: causes and threats. *P T* 40 277–283. 25859123 PMC4378521

[B87] von Salm J. L. Witowski C. G. Fleeman R. M. McClintock J. B. Amsler C. D. Shaw L. N. (2016). Darwinolide, a new diterpene scaffold that inhibits methicillin-resistant *Staphylococcus aureus* biofilm from the antarctic sponge Dendrilla membranosa. *Org. Lett.* 18 2596–2599. 10.1021/acs.orglett.6b00979 27175857 PMC4928490

[B88] Watkins W. J. Landaverry Y. Léger R. Litman R. Renau T. E. Williams N. (2003). The relationship between physicochemical properties, In vitro activity and pharmacokinetic profiles of analogues of diamine-Containing efflux pump inhibitors. *Bioorgan. Med. Chem. Lett.* 13 4241–4244. 10.1016/j.bmcl.2003.07.030 14623009

[B89] Wax R. G. Lewis K. Salyers A. A. Taber H. (2001). *Bacterial Resistance to Antimicrobials.* Boca Raton, FL: CRC Press.

[B90] Webber M. A. Lamers R. P. Cavallari J. F. Burrows L. L. (2013). The efflux inhibitor phenylalanine-arginine beta-naphthylamide (PAβN) permeabilizes the outer membrane of gram-negative bacteria. *PLoS One* 8:e60666. 10.1371/journal.pone.0060666 23544160 PMC3609863

[B91] Weinstein R. A. Hooper D. C. (2005). Efflux pumps and nosocomial antibiotic resistance: a primer for hospital epidemiologists. *Clin. Infect. Dis.* 40 1811–1817. 10.1086/430381 15909271

[B92] Worthington R. J. Melander C. (2013). Combination approaches to combat multidrug-resistant bacteria. *Trends Biotechnol.* 31 177–184. 10.1016/j.tibtech.2012.12.006 23333434 PMC3594660

[B93] Wu J. Zhang Y. Maida L. E. Santos R. G. Welmaker G. S. LaVoi T. M. (2013). Scaffold ranking and positional scanning utilized in the discovery of nAChR-selective compounds suitable for optimization studies. *J. Med. Chem.* 56 10103–10117. 10.1021/jm401543h 24274400 PMC4106133

[B94] Yoneyama H. Katsumata R. (2014). Antibiotic resistance in bacteria and its future for novel antibiotic development. *Biosci. Biotechnol. Biochem.* 70 1060–1075. 10.1271/bbb.70.1060 16717405

[B95] Zelle R. E. (1998). S5726184.

[B96] Zelle R. E. Harding M. W. (1996). US5543423.

