# Supplementary material for: Identification of a Novel Polyamine Scaffold With Potent Efflux Pump Inhibition Activity Toward Multi-Drug Resistant Bacterial Pathogens
Source: Front Microbiol. 2018 Jun 14;9:1301. doi: 10.3389/fmicb.2018.01301 (PMC6010545; doi:10.3389/fmicb.2018.01301)
Supplement: Supplementary file 1 [file Data_Sheet_1.PDF]

Supplementary Material

Identification of a novel polyamine scaffold with potent efflux pump inhibition activity towards multi-drug resistant bacterial pathogens

Renee M. Fleeman*, Ginamarie Debevec, Kirsten Antonen, Jessie L. Adams, Radleigh G. Santos, Gregory S. Welmaker, Richard A. Houghten, Marc A. Giulianotti and Lindsey N. Shaw

*** Correspondence:** Lindsey N. Shaw: shaw@usf.edu

1 Supplementary Data

Chemistry section: General methods

Synthesis of library 2229 and individual compounds and construction of scaffold ranking plate. Library 2229 as well as the individual compounds reported herein (**247**, **250**, **266**, **271**, and **314**) were synthesized following the same synthetic scheme (**Fig. S1**) as previously reported [1-3]. Utilizing the "tea-bag" methodology [4], 100 mg of p-methylbenzhydrylamine (MBHA) resin (1.1 mmol/g, 100-200 mesh) was sealed in a mesh "tea-bag", neutralized with 5% diisopropylethylamine (DIEA) in dichloromethane (DCM) and subsequently swelled with additional DCM washes. Boc-Amino Acids (R_1) were coupled in Dimethylformamide (0.1M DMF) for 120 mins in the presence of Diisopropylcarbodiimide (DIC, 6 equiv.) and 1-Hydroxybenzotriazole hydrate (HOBt, 6 equiv.) (**Step 1, Fig. S1**). The Boc protecting group was then removed with Trifluoroacetic Acid (TFA) in DCM for 30 mins and subsequently neutralized with 5% DIEA/DCM (3x). Boc-Amino Acids (R_2) were coupled utilizing standard coupling procedures (6 equiv.) with DIC (6 equiv.) and HOBt (6 equiv.) in DMF (0.1 M) for 120 mins. The Boc group was removed with 55% TFA/DCM for 30 mins and subsequently neutralized with 5% DIEA/DCM (3x). Carboxylic acids (R_3) were coupled using (10 equiv.) in the presence of DIC (10 equiv.) and HOBt (10 equiv.) in DMF (0.1 M) for 120 mins (**Step 3, Fig. S1**). All coupling reactions were monitored for completion using Ninhydrin. The reduction was performed in a 4000 mL Wilmad LabGlass vessel under nitrogen. 1.0 M Tetrahydrofuran (THF) borane complex solution was used in 40-fold excess for each amide bond. The vessel was heated to 65°C and maintained at this temperature for 96 hrs. The solution was then removed and the bags were washed with THF and methanol (MeOH). Once completely dry, the bags were treated overnight with piperidine at 65°C and washed several times with DMF, DCM and methanol. As previously reported by our group and others, the reduction of polyamides with borane is free of racemization [5-7]. Completion of reduction was monitored by LCMS analysis of a control compound that was cleaved from the solid support (HF, anisole, 0°C 7 hr). The resin was cleaved with HF in the presence of anisole in an ice bath at 0°C for 7 hours. After removal of the HF by gaseous N_2, the products were then extracted from the vessels with 95% acetic acid in water, transferred to scintillation vials, frozen and lyophilized. The compounds were then reconstituted in 50% acetonitrile and water, frozen and lyophilized three more times.

HPLC Purification and NMR (247, 250, 266, 271, and 314)

As previously reported [3] all purifications were performed on a Shimadzu Prominence preparative HPLC system, consisting of LC-8A binary solvent pump, a SCL-10A system controller, a SIL-10AP autosampler, and a FRC-10A fraction collector. A Shimadzu SPD-20A UV detector was used for detection. The wavelength was set at 214 nm during analysis. Chromatographic separations were obtained using a Phenomenex Luna C18 preparative column (5 µm, 150 x 21.5 mm i.d.) preceded by a Phenomenex C18 column guard (5 µm, 15 x 21.2 mm i.d.). Prominence prep software was used to set all detection and collection parameters. The mobile phases for HPLC purification were HPLC grade obtained from Sigma Aldrich and Fisher Scientific. The mobile phase consisted of a mixture of Acetonitrile/water (both with 0.1% formic acid). The initial setting for separation was set at 2% (v/v) Acetonitrile, which was held for 2 mins and the gradient was linearly increased to 20% (v/v) Acetonitrile over 4 mins. The gradient was then linearly increased to 55% (v/v) Acetonitrile over 36 mins. The HPLC system was set to automatically flush and re-equilibrate the column after each run for a total of 4 column volumes. The total flow rate was set to 12 mL/min and the total injection volume was set to 3900 µl. The fraction collector was set to collect from 6 to 40 mins. The corresponding fractions were then combined and lyophilized. The 1H spectra were obtained utilizing the Bruker 400 Ascend (400 MHz). NMR chemical shifts were reported in δ (ppm) using the δ 7.26 signal of CDCl3 (1H NMR).

LCMS analysis

As previously reported [3] the purity and identity of all individual compounds was verified using a Shimadzu 2010 LCMS system, consisting of a LC-20AD binary solvent pump, a DGU-20A degasser unit, a CTO-20A column oven, and a SIL-20A HT autosampler. A Shimadzu SPD-M20A diode array detector was used for detection. A full spectra range of 190-600 nm was obtained during analysis. Chromatographic separations were obtained using a Phenomenex GeminiC18 analytical column (5 µm, 50 x 4.6 mm i.d.) preceded by a Phenomenex C18 column guard (5 µm, 4 x 3.0 mm i.d.). All equipment was controlled and integrated by Shimadzu LCMS solutions software version 3. Mobile phases for LCMS analysis were HPLC grade or LCMS grade obtained from Sigma Aldrich and Fisher Scientific. The mobile phases consisted of a mixture LCMS grade Acetonitrile /water (both with 0.1% trifluoroacetic acid for a pH of 2.7). The initial setting for analysis was set at 5% Acetonitrile (v/v), and then was linearly increased to 95% Acetonitrile over 6 mins. The gradient was then held at 95% Acetonitrile for 2 mins, linearly decreased to 5% over 0.10 mins and held for an additional 1.90 mins. The total run time was equal to 12 mins. The total flow rate was set to 0.5 mL/minute. The column oven and flow cell temperature for the diode array detector was set at 30°C. The autosampler temperature was held at 15°C. 5 µl of compound was injected for analysis.

Chemical synthesis of individual compounds

(S)-N6-methyl-N_2-((S)-2-(phenethylamino)-3-phenylpropyl)hexane-1,2,6-triamine (247)

Using the synthetic approach described in Figure **S1** for the synthesis of **247** was synthesized using the following reagents: Boc-L-Lysine(ClZ) (R1), Boc-L-Phenylalanine (R2), phenylacetic acid (R3). Final crude product was purified by HPLC as described above. 1H NMR (400 MHz,Deuterium Oxice): δ 7.44 (br. s., 5 H) 7.34 (br. s., 5 H) 3.40 (br. s., 3 H) 3.16 (br. s., 1 H) 3.06 (br.s., 3 H) 2.94 (br. s., 3 H) 2.78 (br. s., 2 H) 2.71 (br. s., 4 H) 1.62 (br. s., 3 H) 1.26 (br. s., 3 H). LCMS (ESI+) $C_{24}H_{38}N_4$ m/z 383.31 found [M+H]+:383.20.

(S)-N^2-((S)-2-(phenethylamino)-3-phenylpropyl)butane-1,2,4-triamine (250)

Using the synthetic approach described in Figure **S1** for the synthesis of **250** was synthesized using the following reagents: Boc-L-Asparagine(Xan) (R1), Boc-L-Phenylalanine (R2), phenylacetic acid (R3). Final crude product was purified by HPLC as described above. 1H NMR(400 MHz,Deuterium Oxide):δ ppm 7.44 (br. s., 4 H) 7.33 (br. s., 6 H) 3.59 - 3.68 (m, 1H) 3.39 (br. s., 1 H) 3.05 (br. s., 2 H) 2.90 - 2.99 (m, 1 H) 2.63 - 2.90 (m, 2 H) 1.72 – 1.79 (m, 2H) 1.63 – 1.95 (m, 1 H). LCMS (ESI+) calculated for $C_{21}H_{32}N_4$ m/z 341.28 found [M+H]+: 341.15.

(R)-N^6-methyl-N^2-((S)-2-(phenethylamino)-3-phenylpropyl)hexane-1,2,6-triamine (266)

Using the synthetic approach described in Figure **S1** for the synthesis of **266** was synthesized using the following reagents: Boc-D-Lysine(ClZ) (R1), Boc-L-Phenylalanine (R2), phenylacetic acid (R3). Final crude product was purified by HPLC as described above. 1H NMR (400 MHz,Deuterium Oxide):δ. 7.37 - 7.54 (m, 6 H) 7.31 (br. s., 4 H) 3.40 (br. s., 2 H) 2.95 - 3.07 (m, 6 H) 2.90 (dd, J=13.27, 7.15 Hz, 1 H) 2.71 (br. s., 4 H) 1.64 (br. s., 2 H) 1.31 (br. s., 1 H). LCMS (ESI+) calculated for $C_{24}H_{38}N_4$ m/z 383.31 found [M+H]+: 383.20.

(R)-N^2-((S)-2-(phenethylamino)-3-phenylpropyl)pentane-1,2,5-triamine (271)

Using the synthetic approach described in Figure **S1** for the synthesis of **271** was synthesized using the following reagents: Boc-L-Glutamine(Xan) (R1), Boc-L-Phenylalanine (R2), phenylacetic acid (R3). Final crude product was purified by HPLC as described above. 1H NMR (400 MHz,Deuterium Oxide):δ 7.36 - 7.57 (m, 6 H) 7.32 (br. s., 4 H) 3.57 (br. s., 1 H) 3.40 (br.s., 2H) 3.08 - 3.20 (m, 3 H) 3.05 (br. s., 2 H) 2.77 (br. s., 1 H) 1.62 (br.s., 1H) . LCMS (ESI+) calculated for $C_{22}H_{34}N_4$ m/z 354.28found [M+H]+: 355.20.

(S)-N^1-((S)-1-amino-3-phenylpropan-2-yl)-N^2-phenethylpentane-1,2,5-triamine (314)

Using the synthetic approach described in Figure **S1** for the synthesis of **314** was synthesized using the following reagents: Boc-L-Phenylalanine (R1), Boc-L-Glutamine(Xan) (R2), phenylacetic acid (R3). Final crude product was purified by HPLC as described above. 1H NMR(400 MHz,Deuterium Oxide):δ. 7.44 (br. s., 4 H) 7.28 - 7.39 (m, 6 H) 3.29(d, J=6.24 Hz, 1 H) 3.08 (d, J=12.84 Hz, 3 H) 3.00 (br. s., 4 H) 2.75 - 2.95 (m, 4 H) 1.66 (d, J=7.70 Hz, 2 H) LCMS (ESI+) calculated for $C_{22}H_{34}N_4$ m/z 355.28 found [M+H]+:355.20.

2 Supplementary Figures and Tables

2.1 Supplementary Figures

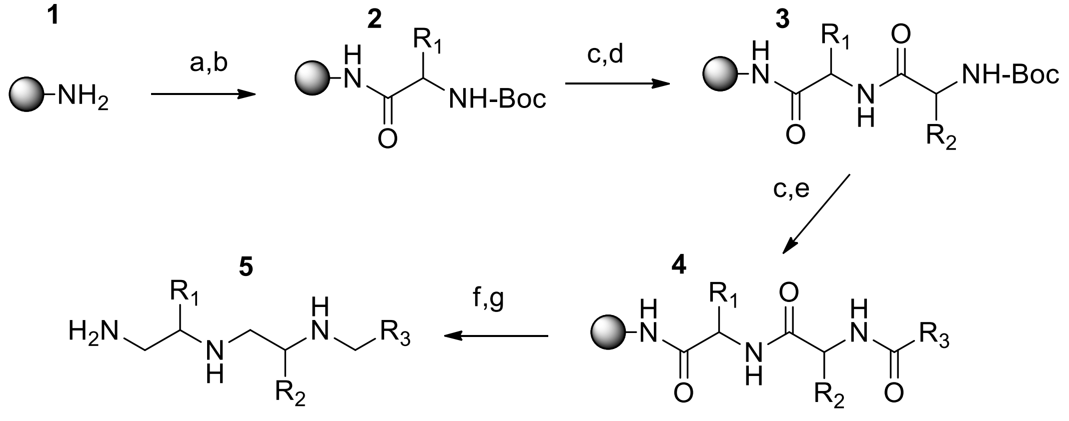

Figure S1. Synthetic Scheme of Polyamines. *a,* 5% DIEA/DCM; *b,* Boc-AA(R_1), DIC, HOBt, DMF; *c,* 55% TFA/DCM; 5% DIEA/DCM; *d,* Boc-AA(R_2), DIC, HOBt, DMF; *e,* R_3COOH, DIC, HOBt, DMF; *f,* BH_3-THF, 65˚C, 96 hours; Piperidine, 65˚C, 24 hours; *g,* HF, anisole, 0˚C.

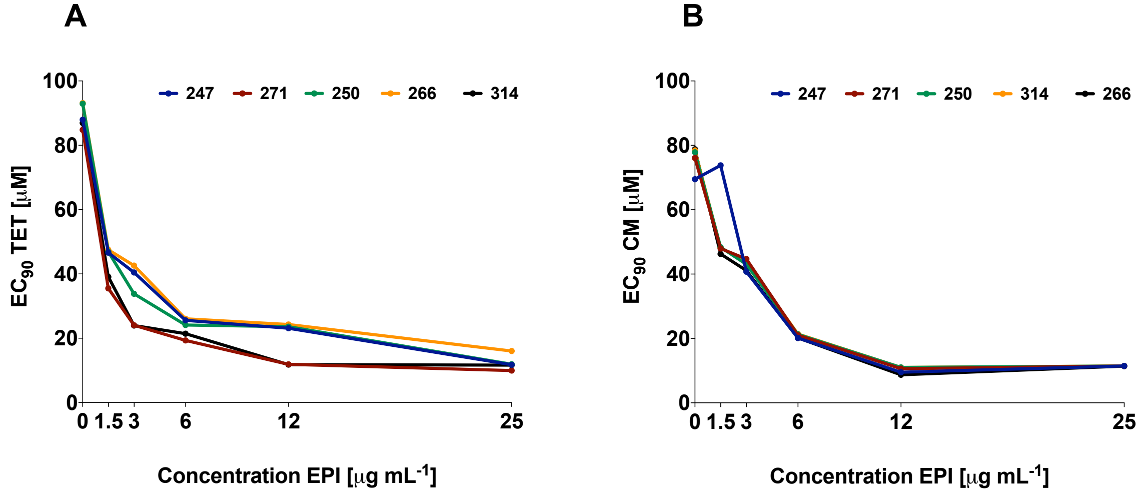

Figure S2. Front runner polyamines potentiate the activity of unrelated antibiotic efflux substrates. *P. aeruginosa* (1419) cells were treated with polyamine agents at increasing concentrations, alongside tetracycline (A) or chloramphenicol (B). Shown is the potentiated EC_{90} concentrations for each antibiotic at increasing EPI concentrations.

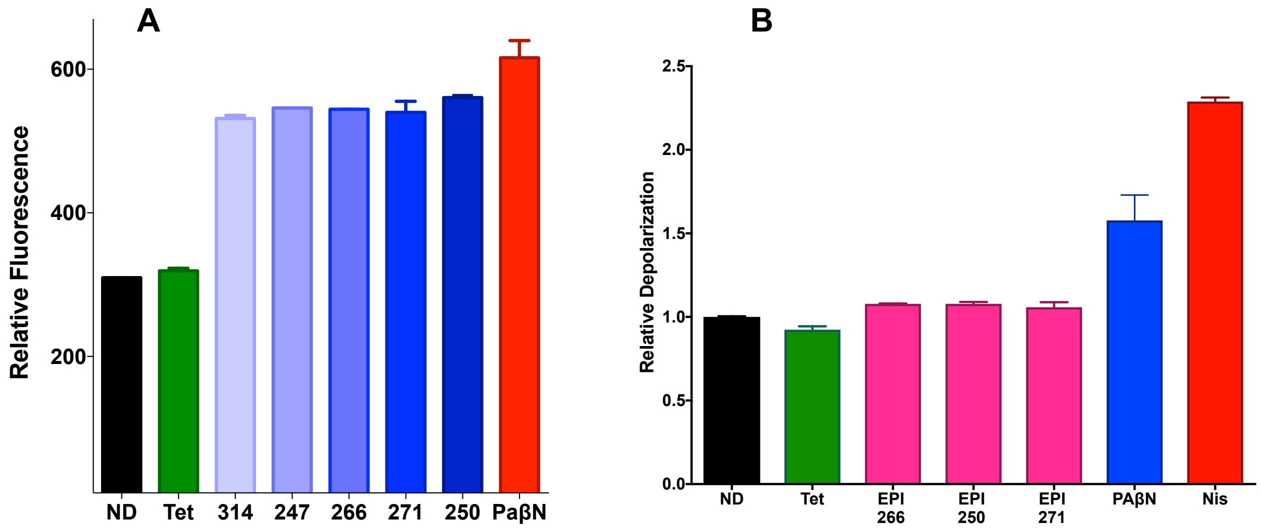

Figure S3. Polyamine Molecules Have Broad Spectrum EPI Activity. (**A**) *S. aureus* (USA 100) cells were treated with a sub-lethal concentration of ethidium bromide (25 µM) in combination with no drug controls (ND), tetracycline (negative control; 25 µM), the known efflux inhibitor PaβN (25 µg mL^{-1}), or lead polyamine agents (25 µg mL^{-1}). Graphs demonstrate fluorescence after 90-minute exposure displayed as relative fluorescent units. (**B**) Relative membrane destabilization compared to no drug (ND) control of the lead polyamines, PaβN, and tetracycline, all at the same concentrations used in **A**. A positive control for *S. aureus* membrane disruption (nisin, 10 µM) was used. All error bars are shown ±SEM.

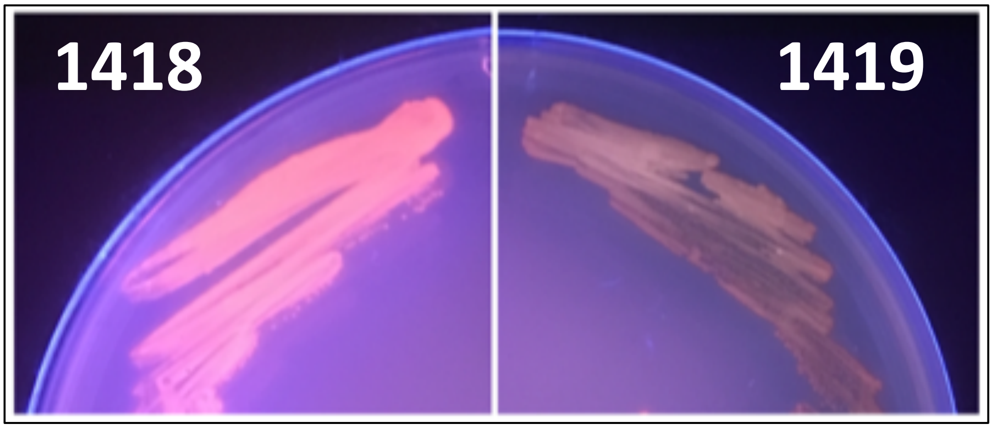

Figure S4. Efflux proficiency of test *P. aeruginosa* strains. These assays were performed as described previously [8]. Specifically, ethidium bromide agar (2 µg mL^{-1}) plates were prepared and inoculated with *P. aeruginosa* clinical isolates. Ethidium bromide intercalates into DNA and fluoresces when exposed to UV light. This compound is a primary efflux pump substrate, thus, those strains that fluoresce are efflux deficient (1418), and those that do not are efflux active (1419).

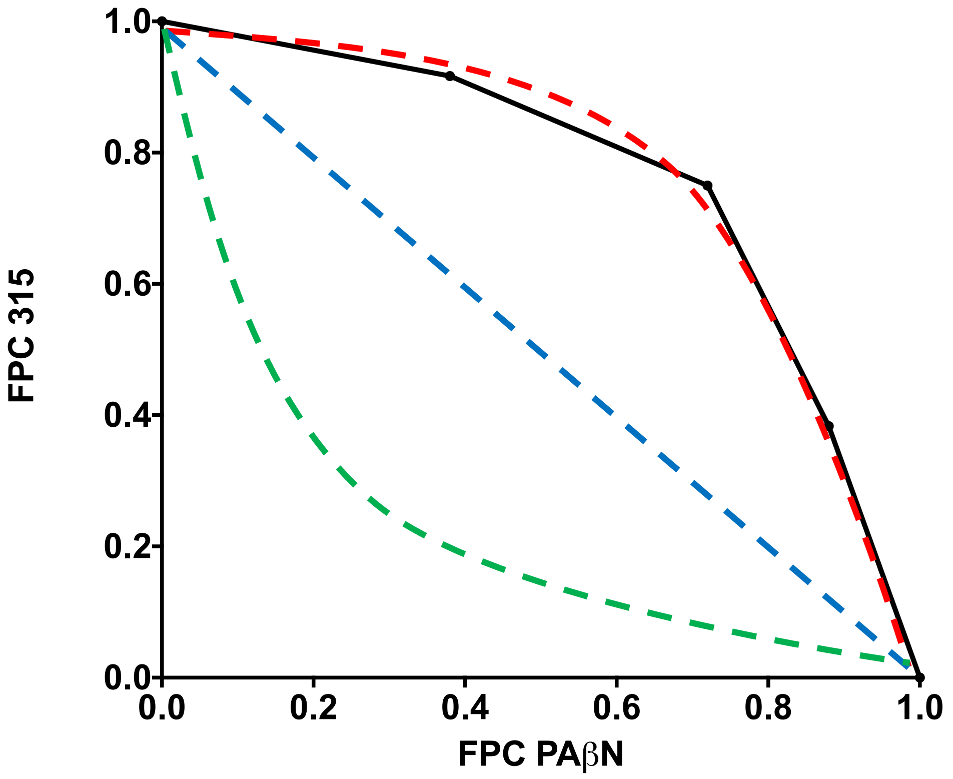

Figure S5. Polyamine molecules are antagonistic to the activity of PAβN. Depicted are hypothetical (dotted lines) and experimentally determined (solid line) representations of EPI synergy. In the experimental conditions (solid black line) *P. aeruginosa* (1419) cells were incubated with increasing concentrations of lead polyamine 315 and PAβN in the presence of a fixed concentration (25 µM) of tetracycline in checkerboard assays. EC_{90} values were determined and used to calculate the fractional potentiating concentration (FPC) of 315 and PAβN (FPC = combination potentiating concentration / potentiating concentration alone). Shown for comparison are the hypothetical shapes of typical experimental curves, indicating synergistic (green), additive (blue), or antagonistic (red) activity for drug combinations.

2.2 Supplementary Tables

Table S1. Clinical isolates used in this study. The table displays all strains used in this study and their origin along with all known genotypes or characteristics. The minimal inhibitory concentrations (μM) for each isolate for tetracycline (TET), chloramphenicol (CHM), aztreonam (ATM), and levofloxacin (LEVO). NT = Not Tested, indicating the organism and drug combination were not used in this study.

Strain name	Genotype or Characteristics	TET	CHM	ATM	LEVO	References
S. aureus						
JE-2 parental	USA300, SCC*mec*IV, pvl+	2.25	110	NT	NT	Fey et al. (2013)[9]
JE-2 *norA* knockout	USA300, SCC*mec*IV, pvl+, *ΔnorA*	NT	100	NT	NT	Fey et al. (2013)[9]
635	USA100	1.76	110	NT	NT	Carroll et al. (2013)[10]
A. baumannii						
1643		NT	85	NT	100	Jacobs et al. (2010)[11]
P. aeruginosa						
1419		100	150	1	NT	Fleeman et al. (2015)[12]
1418		200	>618.94	6	NT	Fleeman et al. (2015)[12]

Table S2. Potentiation assessment of combinatorial scaffold libraries. All data pertains to 90% effective concentration (EC_{90}), which is defined as a 90% decrease in the optical density values for cultures when read at 600nm compared to untreated controls. The **TPL** column refers to the inhibitory concentration of the Torrey Pines Library alone (µg ml^{-1}) to produce an EC_{90} value (note that 25 µg ml^{-1} was the highest concentration tested in these assays). **TET+TPL** displays the tetracycline concentration (µM) required to produce an EC_{90} in the presence of 25 µg ml^{-1} of each library. Fold Potentiation **(FP)** = EC_{90} tetracycline concentration with no TPL / EC_{90} tetracycline concentration with TPL. All assays were performed in triplicate alongside no drug controls, tetracycline alone controls (data not shown due to repetitive values, average concentration for EC_{90} across all replicates = 82.5 µM), as well as positive and negative controls (PaβN, 10% DMF).

LIBRARY	TPL	TET+TPL	FP
**10%DMF**	>25	47.58	1.33
**PABN**	>25	11.63	8.01
**2408**	7.67	3.20	25.63
**2407**	16.74	5.59	15.68
**2353**	15.73	5.93	14.75
**2354**	15.05	23.70	4.22
**2157**	16.15	20.13	4.19
**2229**	>25	21.03	4.18
**2355**	15.17	23.70	3.89
**1955**	>25	23.26	3.57
**2356**	14.37	21.53	2.79
**1954**	>25	32.39	2.69
**2161**	>25	38.32	2.36
**2221**	>25	47.63	2.10
**2048**	>25	47.78	2.09
**2340**	>25	48.71	2.05
**1275**	>25	48.94	2.04
**2304**	>25	48.96	2.04
**2222**	>25	44.76	2.03
**2135**	>25	46.10	1.99
**2225**	>25	46.53	1.99
**2339**	>25	47.58	1.90
**2291**	>25	49.74	1.89
**2443**	>25	46.95	1.88
**1952**	>25	45.08	1.88
**1666**	>25	44.54	1.88
**2220**	>25	49.22	1.86
**2159**	>25	48.83	1.85
**1509**	>25	49.11	1.83
**1295**	>25	47.99	1.82

**2344**	>25	49.95	1.80
**2435**	>25	48.94	1.79
**2228**	>25	49.86	1.79
**2390**	>25	48.43	1.78
**2227**	>25	46.61	1.78
**2439**	>25	47.84	1.76
**1456**	>25	45.46	1.76
**2160**	>25	48.13	1.76
**2352**	>25	49.82	1.76
**1953**	>25	48.71	1.75
**2057**	>25	49.24	1.74
**2058**	>25	48.45	1.74
**2388**	>25	49.13	1.74
**1988**	>25	48.48	1.73
**1481**	>25	50.55	1.72
**1409**	>25	49.04	1.71
**1989**	>25	48.56	1.68
**1983**	>25	48.27	1.66
**2068**	>25	48.77	1.66
**1984**	>25	49.46	1.65
**2321**	>25	48.20	1.65
**2320**	>25	52.74	1.62
**2158**	>25	49.17	1.61
**1277**	>25	49.76	1.59
**2049**	>25	48.45	1.58
**1387**	>25	47.01	1.57
**1276**	>25	56.20	1.56
**2103**	>25	58.83	1.54
**1661**	>25	47.95	1.53
**2211**	>25	47.22	1.47
**1665**	>25	46.98	1.47
**2226**	>25	48.85	1.45
**2137**	>25	47.90	1.44
**2210**	>25	68.51	1.41
**2239**	>25	68.52	1.39
**882**	>25	72.05	1.39
**2079**	>25	50.47	1.39
**2017**	>25	47.38	1.35
**2337**	>25	46.66	1.33
**2275**	>25	46.09	1.32
**2348**	>25	72.41	1.26
**2069**	>25	80.30	1.19
**2391**	>25	82.32	1.16
**2327**	>25	84.60	1.14

**1324**	>25	89.11	1.12
**1978**	>25	86.78	1.11
**2198**	>25	46.75	1.08
**2338**	>25	44.73	1.08
**2123**	>25	93.46	1.07
**1956**	>25	93.63	1.07
**1662**	>25	95.00	1.05
**2165**	>25	94.07	1.05
**1664**	>25	88.13	1.04

Table S3. Potentiation assessment of a polyamine-derived library of compounds. All data pertains to either 50% effective concentrations (EC_{50})*, or 90% effective concentrations (EC_{90})#, which are defined as a 50% or 90% decrease, respectively, in the optical density values for cultures when read at 600nm, compared to untreated controls. **TPI** columns refer to the inhibitory concentration of the polyamines alone (µg ml^{-1}) to produce EC values (note that 25 µg ml^{-1} was the highest concentration tested in these assays). **TET+TPI** display the tetracycline concentration required to produce EC values (µM) in the presence of 25 µg ml^{-1} of the respective TPI. Fold Potentiation **(FP)** = EC tetracycline concentration with no TPI / EC tetracycline concentration with TPI. All assays were performed in triplicate alongside no drug controls, tetracycline alone controls (data not shown due to repetitive values, average concentration for EC_{50} across all replicates = 47.75 µM; EC_{90} = 82.5 µM) and the original 2229 TPL. TPI polyamines chosen as lead agents are underlined. The table list polyamines that do not have antibacterial activity alone above the compounds that have inhibitory concentrations themselves. Following this segregation, the compounds were ordered by fold potentiation of 90% effective concentration (FP#).

TPI	TPI	FP*	TET+TPI*	FP#	TET+TPI#
**414**	>25	18.2	4	16.4	6
<u>**271**</u>	>25	8.2	5	8.5	10
**393**	>25	6.4	9	8	12
**338**	>25	10.7	5	7.9	12
<u>**250**</u>	>25	7	9	7.8	12
<u>**247**</u>	>25	5	9	7.5	12
<u>**314**</u>	>25	5	9	7.5	12
<u>**266**</u>	>25	6.8	9	5.8	16
**453**	>25	7	6	5.7	15
**348**	>25	8.9	9	5.4	19
**334**	>25	4.9	10	5.1	18
**312**	>25	7.8	9	4.8	20

**370**	>25	4.6	10	4.3	22
**465**	>25	7.4	10	4.3	23
**299**	>25	3.1	15	4.2	24
**333**	>25	6.9	10	4.2	24
**388**	>25	4.4	17	4.2	24
**435**	>25	4.7	16	4.2	23
**306**	>25	6	11	4.1	23
**331**	>25	6.4	11	4.1	24
**346**	>25	5.5	11	4.1	23
**289**	>25	4.4	18	4	25
**326**	>25	4.3	11	4	23
**288**	>25	4.1	17	3.9	24
**297**	>25	3.6	19	3.9	25
**290**	>25	2.9	18	3.7	25
**296**	>25	4.2	11	3.7	24
**408**	>25	5.2	19	3.6	27
**350**	>25	3.3	17	3.4	27
**351**	>25	120.9	0	3.3	24
**362**	>25	3.4	14	3.3	28
**295**	>25	2.6	18	3.1	29
**372**	>25	1	5	3	33
**291**	>25	7.1	9	2.9	34
**284**	>25	6.1	9	2.8	35
**369**	>25	2.5	20	2.4	39
**449**	>25	3.6	21	2.4	41
**300**	>25	4.8	9	2.3	39
**347**	>25	3.5	22	2.3	44
**374**	>25	2.6	20	2.3	39
**451**	>25	4.9	17	2.3	43
**353**	>25	2.8	22	2.2	43
**357**	>25	2.3	20	2.2	43
**358**	>25	2.4	21	2.2	42
**396**	>25	2.8	26	2.2	45

**427**	>25	3.5	21	2.2	43
**461**	>25	2.6	20	2.2	42
**253**	>25	7.5	5	2.1	45
**292**	>25	3.8	20	2.1	47
**395**	>25	2.2	35	2.1	48
**399**	>25	2.7	37	2.1	48
**424**	>25	2.7	37	2.1	48
**432**	>25	2.8	36	2.1	48
**446**	>25	3.1	22	2.1	46
**447**	>25	2.8	35	2.1	48
**294**	>25	3.2	17	2	47
**298**	>25	1.9	24	2	45
**305**	>25	2	35	2	48
**313**	>25	2.2	34	2	49
**319**	>25	2.1	34	2	48
**354**	>25	2.3	28	2	47
**361**	>25	2.1	24	2	46
**365**	>25	2.5	22	2	45
**373**	>25	2.1	23	2	44
**380**	>25	2.1	35	2	48
**382**	>25	2.2	21	2	46
**386**	>25	1.9	29	2	47
**409**	>25	2	37	2	49
**417**	>25	1	6	2	50
**420**	>25	2	38	2	49
**423**	>25	2.7	37	2	49
**433**	>25	2	37	2	50
**437**	>25	2.1	23	2	46
**441**	>25	1.9	38	2	49
**303**	>25	1.8	30	1.9	47
**304**	>25	1.8	33	1.9	49
**327**	>25	1.8	36	1.9	48
**335**	>25	1.4	36	1.9	47

**378**	>25	1.7	30	1.9	49
**383**	>25	1.9	22	1.9	44
**389**	>25	1.4	37	1.9	49
**397**	>25	1.8	28	1.9	49
**436**	>25	1.9	38	1.9	52
**442**	>25	2.1	39	1.9	52
**252**	>25	5.8	10	1.8	50
**285**	>25	6.9	5	1.8	52
**309**	>25	1.1	33	1.8	49
**310**	>25	1.6	26	1.8	49
**311**	>25	1.3	31	1.8	46
**317**	>25	5.6	8	1.8	55
**429**	>25	1.6	35	1.8	49
**301**	>25	1.2	31	1.7	50
**343**	>25	1.1	34	1.7	47
**445**	>25	1.3	32	1.7	49
**463**	>25	2.7	38	1.7	59
**293**	>25	5	9	1.6	61
**307**	>25	1.3	28	1.5	48
**316**	>25	1.7	32	1.5	66
**342**	>25	1.3	37	1.5	60
**377**	>25	1.4	38	1.5	63
**375**	>25	1.5	24	1.4	45
**425**	>25	1.9	39	1.4	72
**321**	>25	1.8	39	1.3	75
**330**	>25	1.7	37	1.3	74
**345**	>25	1.8	34	1.3	72
**387**	>25	2.1	37	1.3	77
**457**	>25	1.8	41	1.3	78
**464**	>25	2.4	41	1.3	77
**318**	>25	1.2	32	1.2	62
**329**	>25	1.7	43	1.2	83
**341**	>25	1.8	42	1.2	80

**355**	>25	1.7	44	1.2	86
**401**	>25	1.6	42	1.2	85
**418**	>25	2.4	42	1.2	86
**438**	>25	1.6	42	1.2	80
**450**	>25	1.9	44	1.2	85
**458**	>25	1.9	42	1.2	83
**308**	>25	4	16	1.1	87
**323**	>25	1.1	67	1.1	93
**324**	>25	1.1	67	1.1	93
**325**	>25	1.3	55	1.1	91
**332**	>25	1	23	1.1	93
**336**	>25	1.8	21	1.1	44
**337**	>25	1.6	45	1.1	88
**339**	>25	1.1	73	1.1	95
**340**	>25	1.2	65	1.1	93
**356**	>25	1.1	65	1.1	93
**363**	>25	1.4	59	1.1	92
**364**	>25	1.2	63	1.1	93
**371**	>25	1.6	48	1.1	89
**379**	>25	1.4	55	1.1	91
**381**	>25	1.5	33	1.1	81
**400**	>25	2.2	46	1.1	89
**402**	>25	2	49	1.1	90
**411**	>25	1.7	45	1.1	87
**412**	>25	1.6	48	1.1	89
**416**	>25	1.4	71	1.1	94
**426**	>25	1.5	66	1.1	94
**428**	>25	1.3	56	1.1	91
**431**	>25	2.1	49	1.1	89
**434**	>25	1.4	66	1.1	94
**439**	>25	1.6	63	1.1	93
**440**	>25	1.8	55	1.1	91
**443**	>25	1.2	67	1.1	94

**444**	>25	1.8	45	1.1	87
**448**	>25	1.9	51	1.1	90
**455**	>25	1.5	65	1.1	93
**456**	>25	1.5	66	1.1	93
**459**	>25	1.2	67	1.1	93
**315**	>25	6.7	6	1	74
**419**	>25	1.2	65	1	93
**403**	18	113.3	1	120.9	1
**407**	17	80.8	1	62.9	2
**391**	9	27.3	1	20.3	2
**385**	16	17.9	3	17.5	5
**376**	4	142.1	0	11.9	4
**430**	15	13.8	5	10.5	9
**384**	4	142.5	0	10.3	5
**287**	22	8.6	5	9	9
**460**	14	11.1	7	8.4	12
**422**	14	9	8	8.3	11
**404**	11	8.3	9	8.3	11
**421**	15	8.8	6	8	11
**454**	14	9.5	6	8	12
**462**	14	9.5	6	8	12
**390**	22	5.1	9	7.5	12
**286**	22	8.5	7	5.9	17
**452**	18	7.7	10	4.9	21
**272**	24	7.7	9	4.6	20
**410**	18	9.9	10	4.5	22
**394**	25	4.9	10	4.1	22
**415**	21	5.3	19	4.1	25
**349**	14	6.7	9	4.1	23
**398**	11	4.2	11	4.1	21
**328**	1	4.8	8	4.1	12
**366**	19	2.5	19	3.7	25
**405**	25	7.2	10	3.4	27

**359**	25	2	19	2.8	24
**406**	18	3.8	20	2.6	36
**392**	15	3.3	10	2.4	20
**367**	2	1.7	19	2.3	32
**413**	19	3.6	19	2	47
**368**	6	3	11	1.9	25
**352**	3	2.3	15	1.8	27
**269**	23	9.7	5	1.5	66
**360**	9	1.7	20	1.3	38
**320**	24	1.9	20	1.2	42
**344**	10	1.5	23	1.1	46

3 References

1. Nefzi A, Giulianotti MA, Houghten RA: **Solid-phase synthesis of bis-heterocyclic compounds from resin-bound orthogonally protected lysine**. *Journal of combinatorial chemistry* 2001, **3**(1):68-70.
2. Nefzi A, Ostresh JM, Houghten RA: **The Current Status of Heterocyclic Combinatorial Libraries**. *Chem Rev* 1997, **97**(2):449-472.
3. Sandhaus S, Annamalai T, Welmaker G, Houghten RA, Paz C, Garcia PK, Andres A, Narula G, Rodrigues Felix C, Geden S *et al*: **Small-Molecule Inhibitors Targeting Topoisomerase I as Novel Antituberculosis Agents**. *Antimicrobial Agents and Chemotherapy* 2016, **60**(7):4028-4036.
4. Houghten RA: **General method for the rapid solid-phase synthesis of large numbers of peptides: specificity of antigen-antibody interaction at the level of individual amino acids**. *Proc Natl Acad Sci U S A* 1985, **82**(15):5131-5135.
5. Ostresh JM, Schoner CC, Hamashin VT, Nefzi A, Meyer JP, Houghten RA: **Solid-Phase Synthesis of Trisubstituted Bicyclic Guanidines via Cyclization of Reduced N-Acylated Dipeptides**. *The Journal of Organic Ohemistry* 1998, **63**(24):8622-8623.
6. Nefzi A, Ostresh JM, Houghten RA: **Parallel solid phase synthesis of tetrasubstituted diethylenetriamines via selective amide alkylation and exhaustive reduction of N-acylated dipeptides**. *Tetrahedron* 1999, **55**(2):335-344.
7. Manku S, Laplante C, Kopac D, Chan T, Hall DG: **A mild and general solid-phase method for the synthesis of chiral polyamines. Solution studies on the cleavage of borane-amine intermediates from the reduction of secondary amides**. *The Journal of organic chemistry* 2001, **66**(3):874-885.
8. Martins M, McCusker MP, Viveiros M, Couto I, Fanning S, Pages JM, Amaral L: **A Simple Method for Assessment of MDR Bacteria for Over-Expressed Efflux Pumps**. *Open Microbiol J* 2013, **7**:72-82.
9. Fey PD, Endres JL, Yajjala VK, Widhelm TJ, Boissy RJ, Bose JL, Bayles KW: **A genetic resource for rapid and comprehensive phenotype screening of nonessential Staphylococcus aureus genes**. *MBio* 2013, **4**(1):e00537-00512.
10. Carroll RK, Burda WN, Roberts JC, Peak KK, Cannons AC, Shaw LN: **Draft Genome Sequence of Strain CBD-635, a Methicillin-Resistant Staphylococcus aureus USA100 Isolate**. *Genome Announc* 2013, **1**(4).
11. Jacobs AC, Hood I, Boyd KL, Olson PD, Morrison JM, Carson S, Sayood K, Iwen PC, Skaar EP, Dunman PM: **Inactivation of Phospholipase D Diminishes Acinetobacter baumannii Pathogenesis**. *Infection and Immunity* 2010, **78**(5):1952-1962.
12. Fleeman R, LaVoi TM, Santos RG, Morales A, Nefzi A, Welmaker GS, Medina-Franco JL, Giulianotti MA, Houghten RA, Shaw LN: **Combinatorial Libraries As a Tool for the Discovery of Novel, Broad-Spectrum Antibacterial Agents Targeting the ESKAPE Pathogens**. *Journal of medicinal chemistry* 2015.

frontiers
